# Aqueous two-polymer phase extraction of single-wall carbon nanotubes using surfactants†

**DOI:** 10.1039/c9na00280d

**Published:** 2019-07-11

**Authors:** Jeffrey A. Fagan

**Affiliations:** Materials Science and Engineering Division, National Institute of Standards and Technology Gaithersburg MD USA 20899 Jeffrey.fagan@nist.gov

## Abstract

This review details the current state of the art in aqueous two-phase extraction (ATPE) based separations of surfactant dispersed single-wall carbon nanotubes by their chemical species, *i.e.*, (n,m) structure, semiconducting or metallic nature, and enantiomeric handedness. Discussions of the factors affecting each separation, including workflow effects, variations of different surfactant and nanotube materials, and the underlying physical mechanism are presented. Lastly an outlook on the applications of ATPE at bench scale and implementation to larger scales is discussed, along with identification of research directions that could further support ATPE development.

## Abstract 2

Aqueous two-polymer phase extraction is described for its best-practice use to isolate single-wall carbon nanotube species.

## Graphical abstract

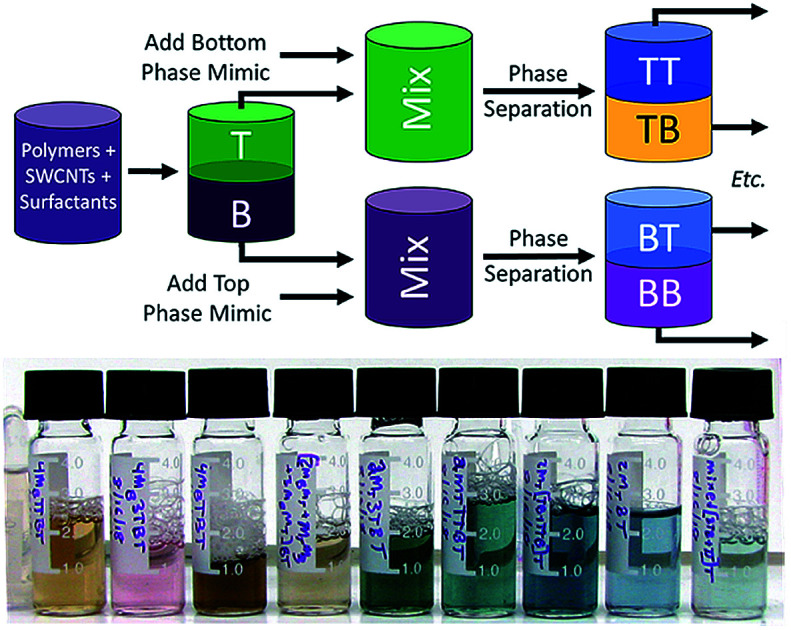

## Introduction

Single-wall carbon nanotubes (SWCNTs) are different from most engineered nanoparticles in that the length scale that primarily determines their confinement-driven properties is set by a distinct crystalline lattice structure for each nanotube species. These species, which are denoted by the (n,m) lattice vector that describes the orientation of the graphene lattice comprising the SWCNT and its diameter, each have unique optical, electrical, thermal and mechanical properties.^1^ However, despite significant and ongoing developments, synthetic methods demonstrated at commercial scale cannot yet synthesize single (n,m) population materials,^2^ but instead produce mixtures of many SWCNT (n,m)s together with impurity carbon forms and residual catalyst. To realize in macroscopic samples the distinct properties of these materials there has subsequently been a decade plus long set of efforts to both purify SWCNTs from non-SWCNT materials,^3–5^ and then to separate that material into subpopulations based on structural characteristics, *e.g.*, diameter, (n,m), enantiomeric handedness, or electronic properties.

Many techniques and separation schemes have been developed to sort heterogenous SWCNT (n,m) and impurity mixtures.^4–6^ For the removal of bulk impurities from SWCNT samples the most common methods are acid treatment for raw SWCNT soot and centrifugation for dispersed SWCNT populations. For subpopulation separations, such as by (n,m), or metallicity, which require prior individualization of the nanotubes and thus a processing step to generate a dispersion, a much more diverse set of methods has been applied including ultracentrifugation (both density gradient (DGU)^7,8^ and rate-zonal methods), electrophoresis,^9^ ion-exchange^10^ and gel chromatography,^11–13^ selective polymer dispersion-extraction in organic solvents^14,15^ and aqueous two-phase extraction (ATPE).^16^ Among the most recently invented and developed techniques, ATPE can be utilized for either bulk purification^17^ or selection of SWCNT subpopulations.^18,19^ In particular, this perspective is focused on the implementation of APTE that utilizes competition of one or more small molecule surfactant types for adsorption on the SWCNT surface to regulate partitioning between the two-phases of a single stage aqueous two-phase system; ATPE separations of SWCNTs driven by selective DNA sequences^20,21^ are a separate processing strategy that is not the primary focus of this contribution.

**Fig. 1:**
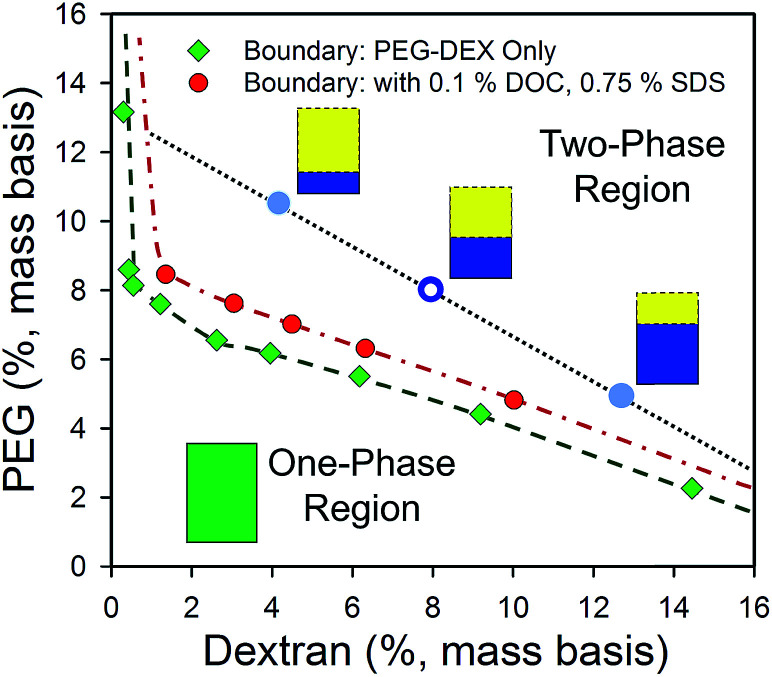
Phase diagram of a 6 kDa PEG and dextran 70 ATPE system in water determined by the cloud point method^22^ without (green diamond symbols) or in the presence of surfactants (red filled circles) at room temperature (≈21 °C). Compositions of PEG and DEX within the two-phase region, such as at the open blue circle, will at equilibrium form two phases at compositions located along the coexistence curve (dashed green eyeguide/dot-dash red eyeguide), and connected by a tie-line (black dotted eyeguide). In the PEG–dextran ATPE system the upper phase is PEG-rich phase, and the lower phase is dextran-rich. All initial compositions along a tie-line produce identical composition of the two phases, but at different volume ratios due to mass conservation (*e.g.* schematic volumes at the blue filled circles). The red circles report the coexistence curve, also called the binodal, of the polymers in the presence of 0.1% (mass basis) DOC and 0.75% (mass basis) SDS, which shifts the coexistence curve to slightly greater polymer mass fractions. Most conditions encountered in the use of surfactant-ATPE for SWCNTs will display similar coexistence curves.

ATPE itself as a separation technique was invented and first developed by Albertsson^22^ in the 1950s for the separation of cellular components and biomolecules. In ATPE, two water-soluble polymers are mixed together in water at a sufficient concentration to drive a thermodynamic phase transition from a single homogenous solution into two phases. Commonly these two polymer phases will have different solution densities, which facilitates coalescence and spatial separation of both phases *via* oppositional sedimentation/creaming within (5 to 20) minutes on the bench or less than ≈ 2 min in a low speed centrifuge. The concentration of the polymers necessary to drive the phase transition depends on the chemical properties of each polymer as well as their molecular mass distributions,^23^ with both the critical point concentrations and phase boundaries well described for many common polymer A:polymer B systems in Albertsson's book^22^ describing the ATPE technique and its uses. For any set of polymer concentrations beyond the critical concentration line, at equilibrium, the mixture will separate into two phases having concentrations at the phase boundaries, one phase rich in polymer A (but with some B) and *vice versa*. The relative volumes of the two phases are determined by the slope of the “tie-line” connecting the two endpoint concentrations and the position of the initial, global, concentration along its length. Importantly, any initial composition of the two polymers along the same tie-line will generate identical compositions of the separated phases, varying only in the relative volume of each phase. The phase diagram for a representative 6 kDa polyethylene glycol (PEG):≈70 kDa dextran (DEX) ATPE system is shown in Fig. 1.

Selective partitioning of additional components, such as solutes or dispersed nanoparticles, occurs if those components have different chemical affinities for the two phases, such that their distribution across the separated polymer phases is not volumetric.^24^ This is the phenomenon enabling separation of SWCNTs by ATPE. We utilize different polymer and additive compositions such that the component SWCNTs to be separated in a mixed population have different affinities for the two phases. In the simplification that the partition coefficients for each component are independent, which is generally reasonable due to their dilute concentration relative to the polymers, the task is then to find the condition, or set of conditions, at which the initial population is best fractionated into the desired subpopulations.^25^

The most common implementation of ATPE for SWCNTs is to use surfactant concentrations to control the SWCNT partitioning between the two phases. The bile salt surfactants sodium deoxycholate (DOC) or sodium cholate (SC) are frequently competed with the alkyl chain structure surfactant sodium dodecyl sulfate (SDS) in a polyethylene glycol (PEG) and dextran (DEX) aqueous two-phase system to control this partitioning. In either case, coverage of the SWCNT sidewall by DOC or SC tends to partition SWCNTs into the bottom, dextran-rich phase, and coverage by SDS partition into the PEG-rich upper phase. Depending on the type of surfactants competed, (n,m) selection (SDS–DOC mostly, with (n,m) selection mainly but not exclusively through diameter selectivity), enantiomeric handedness enrichment, and separation of metallic from semiconducting as well as finer degrees of bandgap size (SDS–SC mostly), have been reported. Different strategies with respect to isolation of purified populations have also been reported; in my lab we tend to apply generalized processing workflows to make several separations with the goal to collect pure, or enriched populations of all (n,m) structures present. Other groups have reported optimized short step routes to isolate specific (n,m) structures,^26–28^ but with the typical cost for such simplification being that all other species are aggregated and discarded. Both strategies can be useful given time constraints and the easier adoption and dissemination of recipe methods, and will be described in more detail in the next section.

Beyond the most common applications of ATPE, it is also readily possible to use other surfactants, 3-component surfactant mixtures, different polymer two-phase systems, and/or many additional modulating chemicals. In our labs at NIST, and in other reports, screening has identified a number of alternative methods that can be applied with varying levels of success and utility. Development of new ATPE variations should also be expected given the relative newness of the method. Although some instances are noted below, the phase space of possible variation is too broad to describe all known or reported variants in this contribution.

## Theory

Partition coefficients, *K*_i_, are defined as the ratio of the concentration of component i in the top phase, *c*_T,i_, to the concentration of the same component in the bottom phase, *c*_B,i_. Assuming the partition coefficient of each (n,m) is independent of both that of other species and its own concentration, 
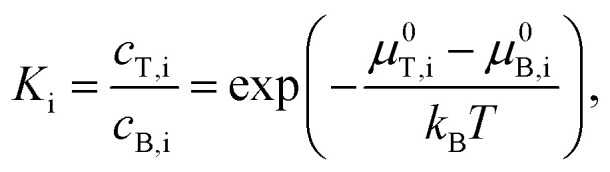
 in which *μ*^0^_T,i_ and *μ*^0^_B,i_ are the standard chemical potentials of component i in the top and bottom phases, *k*_B_ is the Boltzmann constant, and *T* is the absolute temperature.^16^ Thus, relatively small differences in chemical potential of different SWCNT (n,m)s in the two phases can lead to large differences in partitioning, especially if the difference of the top and bottom chemical potentials switches signs. The effectiveness of separation is then also affected by mass conservation, which require *V*_tot_*c*_tot,i_ = *V*_T_*c*_T,i_ + *V*_B_*c*_B,i_ + *V*_I_*c*_I,i_, in which *V*_T_, *V*_B_, and *V*_I_ are, respectively, the volumes of the top phase, bottom phase and the interface. This is worth remembering because large ratios in the volumes of the two polymer phases can constrain the effectiveness of separation of two (n,m) species if the partition coefficients are not reasonably different.

Whether the partitioning of the species reaches equilibrium before the two polymer phases spatially separate due to mass transfer limitations, *i.e.* can the SWCNTs distribute across the separating two-phase chemical gradient fast enough to reach their desired equilibrium distribution before the spatial separation of the coalescing polymer phases makes it impossible, also raises the issue of stage efficiency and the concept of theoretical plates of separation. From a practical standpoint, mass transfer effects tend to be biased against the transfer of (n,m)s from the phase the SWCNT is in to the other phase, *i.e.*, against SWCNTs in the bottom phase partitioning into a top phase in a sequential ATPE step, so excepting applications such as chromatographic separation (*vide infra*), the analogy to separations such as distillation is not complete. However, the fact that non-achievement of equilibrium will also limit the realized separation should be remembered.

## Discussion

The utility of the ATPE separation method as compared to other techniques previously used for SWCNT sorting is immense for several reasons. These include the high tunability of the separation, the rapid and efficient partitioning by spinodal decomposition of the phases, and the ease of accomplishing both separation of semiconducting from metallic SWCNTs and diameter separation in the same chemical system. Additional practical benefits are the need for no major equipment, low energy costs, the nontoxicity and low cost of the polymers, and much greater mass throughput than DGU or gel chromatography. Variations of this still developing technique have shown applicability across a broad range of SWCNT diameters with minimal adjustment, the capability to separate SWCNTs all the way to the enantiomer level, and even the ability to isolate DWCNT subpopulations.^29^ Here I highlight the strengths and primary variations for SWCNT processing.

### Overview: single and multistage separations

**Fig. 2:**
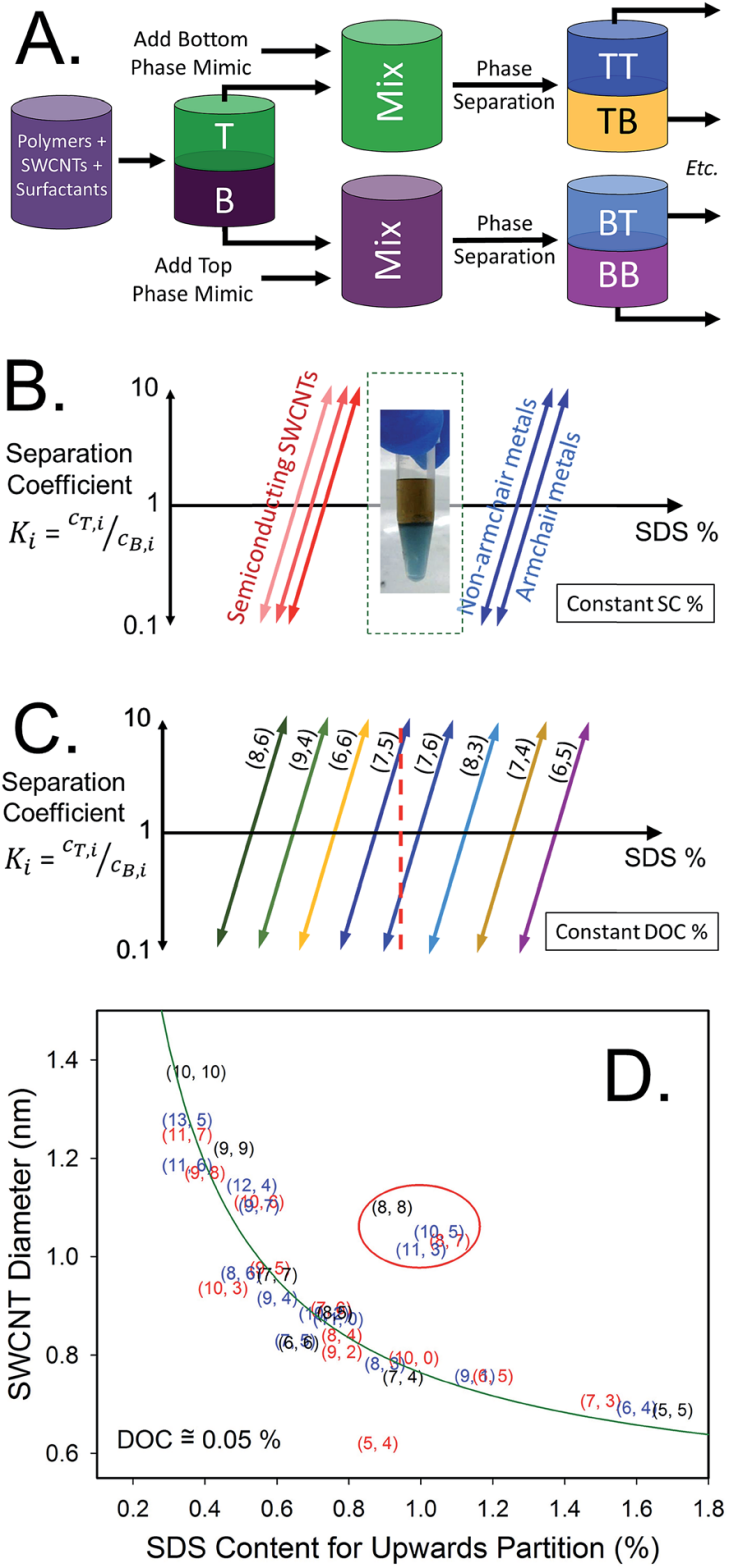
(A) Schematic of a multistage ATPE separation. (B) Schematic of the separation coefficient functionality for a semiconducting–metallic SWCNT separation at constant SC and oxidant (NaClO) concentration as a function of SDS addition. (C) Schematic of the separation coefficient functionality of different (n,m) SWCNTs for SDS/DOC competition separations as a function of SDS concentration for constant DOC. At the SDS concentration shown by the red (dashed) vertical line, approximately all of the (8,6), (9,4), (6,6) and most of the (7,5) would partition to the top phase, with the other shown species partitioning to the bottom phase. (D) Points showing the approximate SDS concentration required to partition the labelled species into the top phase of the PEG 6 kDa:DEX 70 ATPE system at ≈20 °C and a DOC concentration of 0.05%. The data quality was insufficient to discriminate enantiomers. Adapted from Fagan *et al.*,^19^*ACS Nano*, 2015, **9**, 5377–5390.

A key ability of ATPE for SWCNT sorting is the option of applying multiple stages of ATPE separation at different, but controlled, conditions to refine separations, and, if necessary, to switch between diameter and semiconducting–metallic separation to reach highly purified fractions in one process. In a single fractionation, *i.e.*, a single stage, some SWCNTs will partition into the top phase, and some into the bottom phase, with the split controlled by the surfactant concentrations within the stage. For multistage separation, the resulting top and bottom phases are first physically separated from each other, usually by pipetting or decanting, and then new polymer and surfactant is added and mixed with the first phase such that another two-polymer phase separation will occur and further refine the fractionating SWCNT population. This is shown schematically in Fig. 2A. In our internal lingo, we successively add a label, *T* or *B*, for each stage to denominate the potentially geometrically increasing number of phases. To keep track of when the goal of the fractionation was semiconducting–metallic SWCNT separation the phases are alternatively labelled with a M_T_ or M_B_, for respective transfer to the top phase (usually semiconducting enriched) or bottom phase (metallic enriched). For instance, a sub-population of SWCNTs that partitioned into the top phase in the first two stages, subjected to a semiconducting–metallic separation in the third stage with partition into the top phase, a normal partition into the bottom phase during the fourth stage, and then again into the top phase would be labelled TTM_T_BT. For samples that repetitively partition to the same phase across multiple sequential stages these labels are collapsed to save space, *e.g.* BBBBT becomes 4BT. I have attempted to avoid using imprecise terminology in this contribution, but terms such as “pushed down”, referring to transferring a subpopulation into a bottom phase or “pulled up”, *i.e.*, transferring a subpopulation into an upper phase are commonly used by practitioners and may have slipped through.

The governing factors for each stage of the separation can be viewed through the lens of the separation coefficient. Schematic figures for the separation coefficient for SDS/SC competition and SDS/DOC competition are shown in Fig. 2B and C respectively.

In the semiconducting–metallic separation schematic in Fig. 2B, there is a window of SDS concentrations, at the implied SC and oxidant concentrations, that allows for differential partitioning of the semiconducting and metallic subpopulations of the SWCNT dispersion. At low SDS concentration, all SWCNTs partition into the bottom phase (all *K*_i_ ≪ 1). With increased SDS concentration, first semiconducting SWCNTs will partition to the top phase (*K*_semiconducting_ ≥ 1), followed by metallic SWCNTs at significantly greater SDS concentration. At SDS concentrations in between these two values, separation of the semiconducting and metallic SWCNTs will occur as indicated by the picture (*K*_semiconducting_ ≫ 1, *K*_metallic_ ≪ 1).

In the schematic of Fig. 2C, the competition of SDS and DOC for each SWCNT's surface instead produces a series of separation coefficient curves as a function of SDS concentration. Each SWCNT (n,m), and more precisely its (m,n) enantiomer as well, appears experimentally to have a different separation coefficient functionality. Performing an ATPE separation at any point along the SDS concentration vector in Fig. 2C, and imagining that the separation coefficients are all step functions, all of the SWCNT (n,m)s to the left of the SDS concentration chosen will partition into the top phase, and all the SWCNT (n,m)s to the right into the bottom phase. Empirically the separation coefficient functionality is not a step function, however, and for (n,m) structures with curves that overlap the chosen SDS concentration, those (n,m)s will distribute between both phases at equilibrium. The approximate partition points, where the separation coefficient equals 1, for each SWCNT species were first collated into a figure in Fagan *et al.*^19^ for selected species for which data was available. This figure is reproduced as Fig. 2D. In this approximate, and empirical, figure, it is clear that there is both a significant diameter trend with respect to the SDS concentration at which individual (n,m) species partition, but also that there is significant scatter from the trend on the basis of the specific structure. Better clarification of these values, and with greater species and enantiomer resolution, is a recommendation of this contribution.

With the governing ideas described, the application of different surfactant combinations for different separation schemes and workflows can be presented.

### Purification

**Fig. 3:**
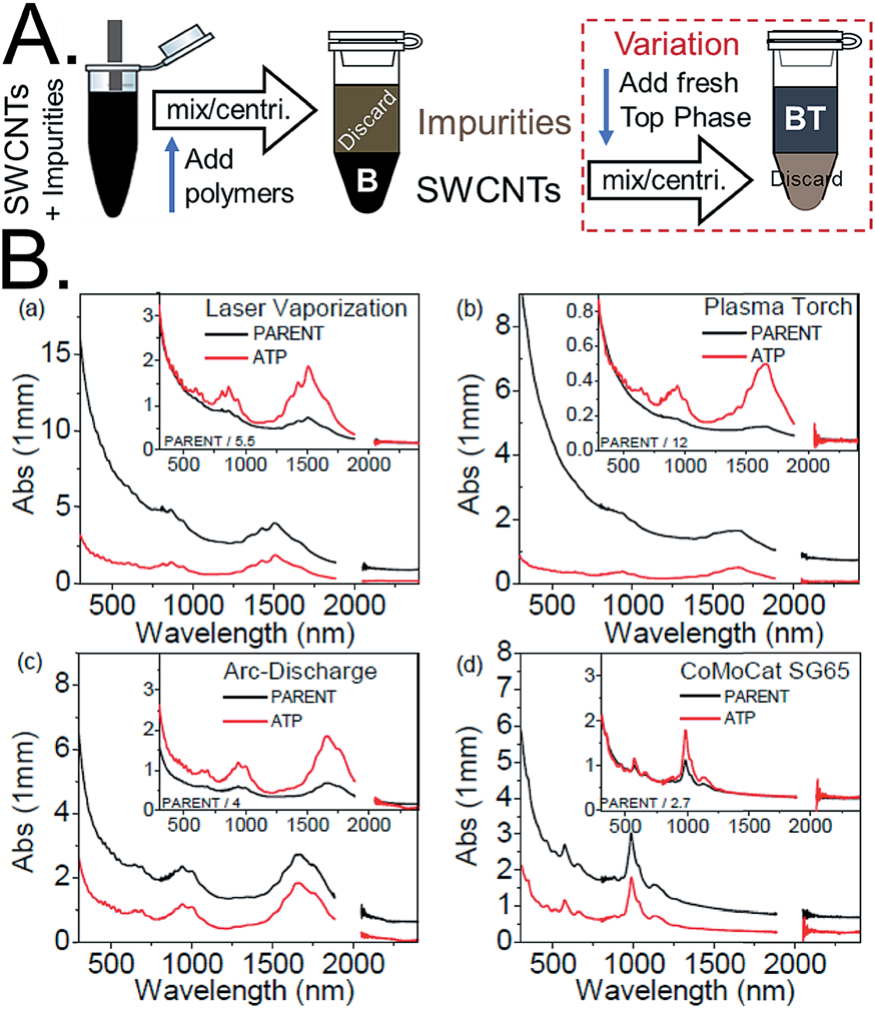
(A) Schematic of SWCNT dispersion purification by ATPE as described by Subbaiyan *et al.*,^17^ and with the variation of adding a second step to transfer the purified dispersion to a top phase for collection. For DOC dispersed SWCNT populations, the DOC concentration is reduced to a quantity that enables partition of many impurity types into the top phase, which can be discarded, while retaining the individualized SWCNTs in the bottom phase. In the variation including a second step, addition of SDS and reduction of the DOC concentration with addition of more top phase polymer, is specified to just at the condition needed to partition all SWCNT species to the top phase. Additional impurities will remain in the bottom phase if done correctly. (B) Figures from Subbaiyan *et al.*^17^ demonstrating the purification of SWCNT populations of several different average diameters that partition to the bottom phase relative to the original dispersion as shown by the reduction in absorbance that does not display the specific SWCNT optical transitions. Reprinted with permission from Springer Nature: Subbaiyan *et al.*, *Nano Res.*, 2015, **8**(5), 1755–1769. Copyright 2014.

A simple but notable use of ATPE was reported by Subbaiyan *et al.*,^17^ which is to take advantage of the differential affinity of many impurities within a SWCNT dispersion for the alternate phase when the surfactant concentration is precisely controlled. They demonstrated that a single (or in practical application two) ATPE steps could accomplish similar purification of the initial dispersion as application of bulk centrifugation and worked for SWCNTs up to at least 20 μm in length. A diagram of their approach is presented as Fig. 3A. For DOC dispersed SWCNTs, their method removes many impurities to the top phase by reducing the DOC concentration to a level insufficient to hold impurities in the bottom phase, but that retains good dispersion and bottom phase affinity for individualized SWCNTs. After this step, in my lab we would add SDS and transfer the individualized SWCNTs to end in the preferred PEG phase (*vide infra*), which can additionally purify the SWCNTs, or one can proceed directly to additional separation steps. Although this is a promising approach, and potential very suitable for large scale applications of ATPE, we have not adopted this methodology in our labs, primarily for reasons of continuity with prior processing of SWCNT samples. However, issues such as jamming at high mass loading of SWCNTs (and associated non-SWCNT impurities in the unpurified dispersion), which can form a thin to thick interfacial-trapped gel layer and reduce the yield of good SWCNTs being able to segregate to their desired phase, and implementing a multistage process of the purification to reduce polymer consumption have yet to be addressed.

In the absence of using highly purified SWCNTs as the ATPE input, however, this purification step can be very valuable, particularly in the isolation of metallic SWCNTs. Many impurities partition similarly to the metallic SWCNTs over the first few stages of the semiconducting–metallic SWCNT separation; removing the contaminants beforehand allows readier visual assessment of that separation.

### Semiconducting from metallic SWCNT separation

The easiest application of ATPE is for the separation of highly enriched semiconducting SWCNT populations from the parent dispersion.^16,30,31^ In my lab this is performed by competing SC with SDS for the SWCNT surface in the ATPE system, most effectively while in the presence of an oxidizing compound such as sodium hypochlorite (NaClO). In the important report of Gui *et al.*,^35^ the authors showed that addition of a small quantity of such an oxidizing agent both vastly improved robustness of the semiconducting from metallic separation fidelity, by effectively setting the redox potential in the ATPE system rather than relying on the ambient level, and could also enable band gap selection, critically between true metallic armchair, (n,m = n), SWCNTs and the *k*_B_*T* level bandgap semi-metallic SWCNTs. Because a SWCNT's band gap is strongly related to its diameter, the optimal amount of SC, SDS and oxidant vary with the average diameter of the SWCNT population to be fractionated. Especially for nanotube populations including small diameter SWCNTs it is a good strategy to perform a single separation by diameter, as reported in the next section, before semiconducting–metallic separation to avoid “cross talk” between the two parameters in the separation results.

**Fig. 4:**
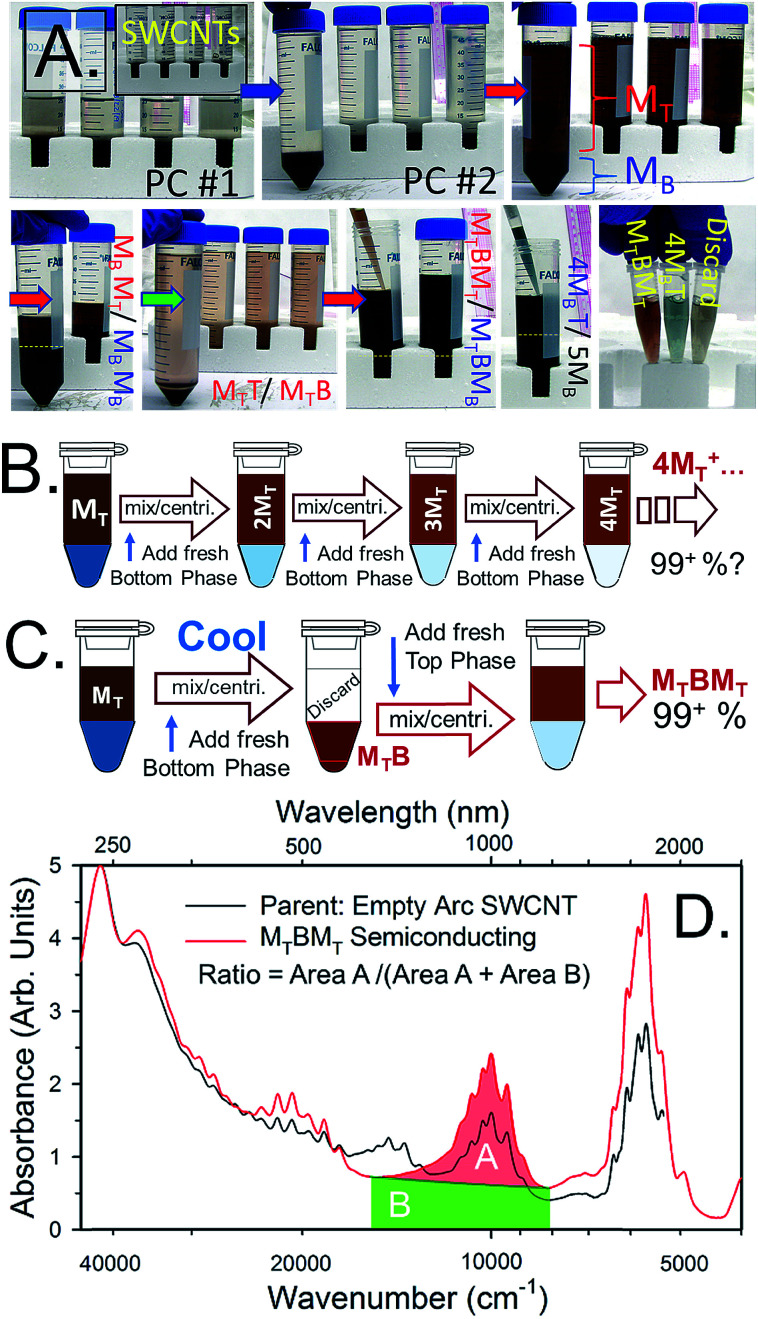
(A) Photographs of water-filled electric arc synthesis SWCNTs being separated on the basis of semiconducting and metallic electrical properties from start to finish. The separation conditions for the semiconducting/metallic separation in the in the 3^rd^, 4^th^ and 6^th^ photos were 0.7% SDS, 0.9% SC and (4 to 5) μL mL^−1^ 1/100^th^ conc. NaClO. After separation, the red-orange-colored semiconducting SWCNTs primarily reside in the top phase, and the blue-green metallic SWCNTs primarily in the bottom phase. The photos are representative of the SWCNT concentration at which the separation is typically conducted in our labs. (B) A possible workflow for increasing semiconducting purity of the top fraction by repeatedly performing ATPE steps to remove contaminant metallic SWCNTs to fresh bottom phase. (C) A recommended replacement workflow in which a temperature change is used to shift all of the SWCNTs into a fresh bottom phase before application of a second, partitioning, ATPE step (back at room temperature). (D) Absorbance spectra of the semiconducting empty electric arc SWCNT synthesis SWCNTs separated as in (A) (red line) compared to the parent dispersion (black line). Absorbance features due to metallic SWCNT such as the peak features around ≈700 nm in the parent dispersion are near completely removed after the separation. The filled areas highlight the integrated regions of the absorbance spectra used for semiconducting purity assessment in literature methods.^15^ The ratio of *A*/(*A* + *B*) as measured over the shown wavenumber range (8110–15 575) cm^−1^ is 0.426.

A series of photographs are shown in Fig. 4A of an example semiconducting–metallic separation using electric arc synthesis, ≈1.5 nm average diameter, SWCNTs. In the first row of photographs, four identical vials containing 10 mL each of SWCNT dispersion are first subjected to two ATPE steps to reduce the concentration of DOC in the DOC-dispersed SWCNT sample and to add SC. In the third photograph, the SWCNT containing phase, marked PC#2 in the second photo, has been mixed with additional surfactant, polymers, and oxidant to reach the conditions at which semiconducting/metallic separation will occur. After centrifugation the semiconducting SWCNTs are primarily in the top phase, and the metallic SWCNTs in the bottom. The semiconducting SWCNTs are red-orange colored and the metallic SWCNTs are blue-green for this diameter mixture. In the second row of photos, extraction of additional semiconducting SWCNTs from the M_B_ population and the separation of high purity semiconducting (M_T_BM_T_) and metallic (4M_B_T) populations are shown. Last in the row is a picture of aliquots of the populations in shorter pathlength vials (and after dilution) to show the color difference. As shown in the photos, the ATPE separation can be conducted at SWCNT concentrations over 0.5 mg mL^−1^. In such cases, a useful tip is to look through the thinner path length of the pipette during extraction to locate the interface. Additional guidance for achieving the best fidelity of separating the interface are described in detail in a later section.

Beyond the surfactant, oxidant, and polymer concentrations of the separation, the order and direction of the ATPE separation can also strongly affect the efficiency of the separation. In Fig. 4B and C two possible workflow diagrams are shown for the separation of a high purity semiconducting SWCNT sample. In general, the workflow in Fig. 4B of sequentially attempting to remove metallic SWCNTs from a semiconducting-enriched top phase over multiple ATPE steps^16,30,32^ is disfavored over the workflow of Fig. 4C even though high purity populations are eventually produced. This is due mostly to kinetic phenomena that prevent the achievement of true equilibrium redistribution of the SWCNTs at each ATPE step. In the workflow of Fig. 4B after the first separation one is essentially trying to remove a small fractional impurity (metallic SWCNTs) from a large volume, but non-achievement of equilibrium (and volumetric effects) tends to retain the contaminant metallics with the semiconducting SWCNTs. The workflow in Fig. 4C is thus favored because, after utilizing a temperature swing to transfer all SWCNTs back to the bottom phase (*vide infra*), both mass transfer factors and thermodynamic equilibrium will act to retain the metallic SWCNTs in the lower phase, resulting in a more rapid semiconducting purity enhancement.

Two notes on implementing either workflow. One, for the ATPE separation of semiconducting from metallic SWCNTs it is critical for the DOC concentration to be less than 0.02%. Otherwise a hybrid or diameter separation will occur. This is a reflection of the much stronger adsorption of DOC than SC onto the SWCNT-solution interface for most SWCNTs. Second, after addition of the oxidant and mixing of the vial it is heuristically valuable to wait 30 s to 1 min, remix, wait another 30 s and only then centrifuge. This leads to better fidelity of the partitioning of metallic SWCNTs to the bottom phase. A hypothesis for this observation is that the doping of the metallic SWCNTs is not instantaneous at the ATPE conditions described, but is sufficiently complete after the short wait. Waiting for brief additional amounts of time does not induce notable further changes to the separation.

Evaluation of semiconducting purity resulting from the ATPE separation can be done using the methods advanced by Finnie *et al.*^15,33^ based on a ratio of absorbance areas for wavelength ranges contributed primarily by semiconducting and metallic SWCNTs.^34^ Based on device work^34^ a ratio of 0.42 indicates at least 99.9% semiconducting purity. In the workflow of Fig. 4B^16,30,32^ very high purities are approached, but only after 6+ steps. Absorbance spectra for electric arc synthesis SWCNTs separated by the 2^nd^ workflow are presented in Fig. 4D. The ratio calculated from the shown spectra is 0.426 for the semiconducting SWCNTs in the M_T_BM_T_ phase. Essentially the semiconducting SWCNTs are all collected, at high purity, after three ATPE separation steps.

**Fig. 5:**
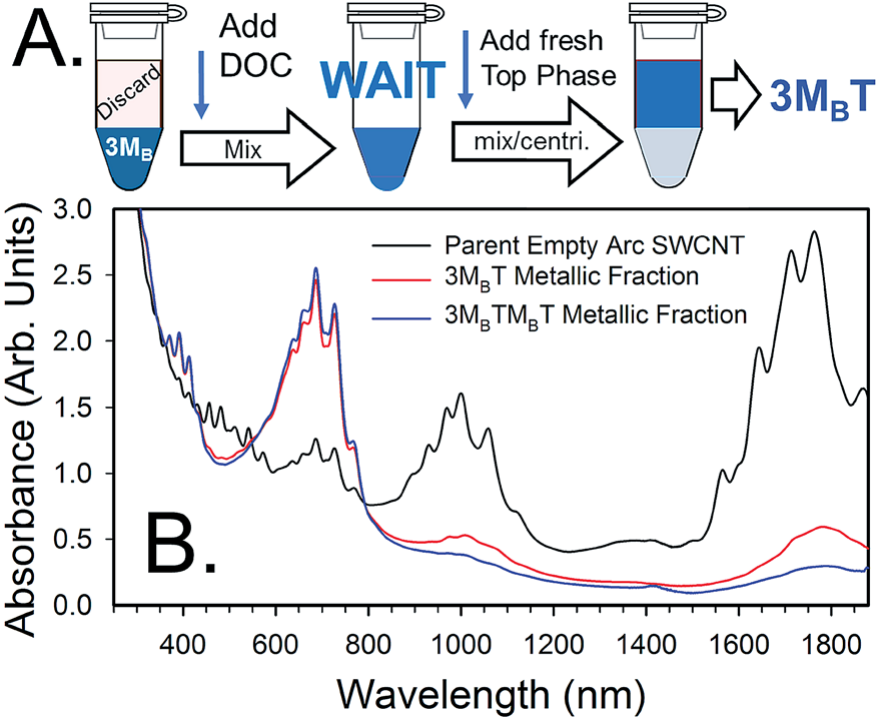
(A) Schematic of additional workflow of ATPE separations for producing high purity metallic SWCNT populations starting at the 3M_B_ (*i.e.* M_B_M_B_M_B_) phase of Fig. 4. After separation of semiconducting SWCNTs to the top phase and their removal *via* pipetting, DOC is added to a concentration of 0.05% in the bottom phase and mixed. After a waiting period of 30 min to 1 h, fresh top phase mimic containing DOC and SDS at a concentration just sufficient to partition all (n,m) species to the top phase is added, mixed and ATPE performed. The last step may need to be repeated on the resultant bottom fraction to collect all of the desirable SWCNTs. (B) Scaled absorbance spectra of metallic-enriched empty arc SWCNTs separated in the manner of workflow in (A) compared to the parent dispersion. The workflow of (A) results in a purer metallic population than achieved without use of DOC; a repetition of the separation further increases metallic purity. Also shown is the effect of re-semiconducting–metallic sorting the DOC-transferred top fraction, followed by a repetition of the workflow. This results in an even greater metallic purity with little recovery loss of the metallic population. Spectra are scaled to an absorbance of A = 5 arb. units at the ≈240 nm peak for all samples.

For metallic SWCNTs, the primary challenge to high purity populations is that oxidized semiconducting SWCNTs and most any remaining impurities partition to the bottom, DEX-rich, phase in the shown steps too. Problematically, simply immediately adding sufficient SDS to shift the desired metallic SWCNTs to the top will also shift some of the contaminants. Also, as discussed above, any factor preventing the ATPE separation from reaching equilibrium will have left some good semiconducting SWCNTs in with the metallic population. To address these factors an additional workflow is presented in Fig. 5A to achieve a high purity of metallic SWCNTs in the top phase.

The key to this workflow in Fig. 5A is to counter the effects of the oxidizing agent (NaClO) on the ATPE separation with a two-pronged strategy. First, DOC is added to 0.05% in the solution, and second, waiting for 30 min to 1 h for the oxidant concentration to diminish. PEG is a weak reducing agent^35^ and will negate the oxidation potential of the NaClO over time; we also hypothesize that as the doping of the SWCNT surface decreases, the DOC forms a tighter surfactant layer on the SWCNT surface blocking re-oxidation. After the incubation period, a top phase with a lesser amount of SDS, but above that used for diameter selection (*vide infra*), will transfer the good metallic SWCNTs to the upper phase. To achieve extreme metallic enrichment in the final population a repetition of a single stage semiconducting/metallic separation followed by reapplication of the workflow in Fig. 5A may be necessary. Results of an example separation in the method of Fig. 5A are shown in Fig. 5B.

**Fig. 6:**
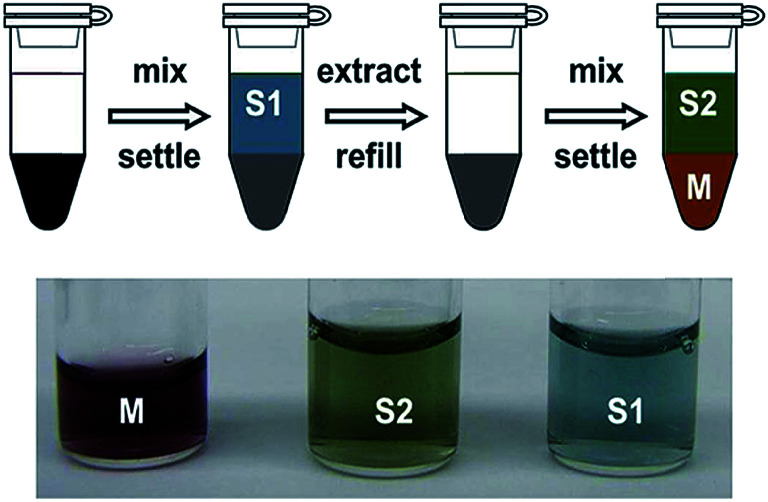
(Top) Schematic of band gap fractionation of a SWCNT sample by sequential ATPE separations at successively reduced oxidant concentrations. (Bottom) Photographs of separated fractions isolated from a small diameter SWCNT population. Adapted with permission from Gui *et al.*, *Nano Lett*., 2015, **15**, 1642–1646. Copyright 2015 American Chemical Society.

Lastly in this section, similar workflows to those shown above have also been shown to enable partitioning on the basis of the SWCNT bandgap in the SDS–SC ATPE system.^35^ Most usefully this can be applied for enriching true, armchair (n,m), metallic SWCNTs from the semi-metallic SWCNT species, but it can also be used to discriminate between species with similar partitioning behaviors in the SDS–DOC ATPE system. For either of these separations it is generally preferred to first transfer all the SWCNTs to the bottom phase by adding oxidant (NaClO). Then the oxidant concentration is slowly reduced *via* multiple remixes with the PEG phase. As discussed above, the PEG slowly reduces the oxidation potential in the solution, such that eventually larger bandgap, followed by smaller bandgap, SWCNTS partition to the upper phase. While starting from either the top phase and adding oxidant or starting from the bottom phase and remixing to reduce its strength, are possible, the slow decreases in oxidation allowed by remixing enables fine differentiation and extraction of the SWCNTs at each stage to the more easily physically removed PEG phase. Together these factors lead to a preference for the bottom phase start methodology. An example of this type of separation is reproduced from Gui *et al.*^35^ in Fig. 6.

### Diameter and (n,m) separation

The second common implementation of ATPE is the competition of SDS and DOC to selectively partition SWCNTs on the basis of their structure. This selection is generally based on diameter, but with significant specific (n,m) scatter and exceptions to a strict diameter order. Described first in Fagan *et al.*,^18^ and expanded upon for larger diameter SWCNTs in Fagan *et al.*,^19^ the order of partition with SDS and DOC content has more recently also been found to be applicable to gel chromatography separations utilizing SDS and DOC implying a unified surface coverage competition mechanism common to both methods.^13^ Thermodynamic studies,^36^ and more detailed resolution of the surfactant competition driven extraction order^37^ are ongoing, but are complicated by spectral congestion and differences in behaviors due to enantiomeric effects.

#### Normal process (gradient, multistep)

The typical implementation of ATPE for diameter–structure separation is the iterative application of ATPE in a multistage cascade. The scheme involves: first, setting the SDS and DOC concentrations to partition multiple (n,m) species into both the resulting top and bottom phases; second, physical separation of the top and bottom phases into separate containers; third, addition of a mimic opposite phase to lower (raise) the SDS concentration of the top (bottom) phase, while holding DOC concentration constant, and reestablishing the global polymer concentrations within the two-phase region; and lastly mixing and separation to split the (n,m)s in each of the original top and bottom fractions into two subpopulations. This multistage work flow was shown schematically in Fig. 2A. As a note, it is helpful for most SWCNT populations to apply a semiconducting–metallic separation step, reducing through dilution the DOC concentration below 0.02% and adding SC to shift the surfactant composition, either before diameter–structure separation or after only one or two ATPE stages. This is a helpful strategy because it efficiently refines the (n,m) species distributions in subsequent stages to sets with similar electronic properties, and, because the semiconducting–metallic separation is mostly diameter agnostic, widens the SDS concentration differences between the partitioning of the remaining differing diameter SWCNT (n,m)s.

**Fig. 7:**
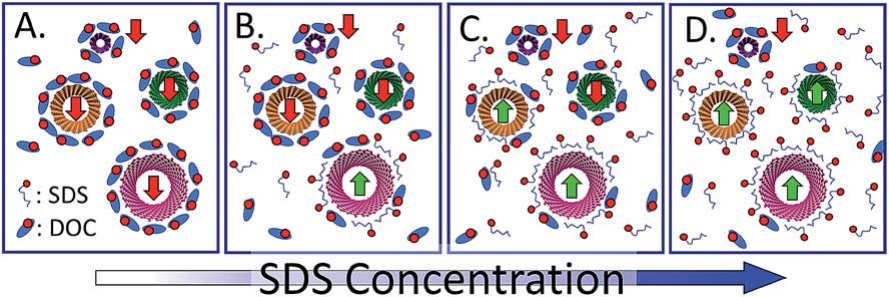
Simplified schematic of the hypothesized mechanism driving the selection of ATPE partition for each individual (n,m) species in the SDS–DOC competition ATPE system for a constant DOC concentration. (A) At low SDS concentration all SWCNT (n,m)s are covered primarily or entirely by DOC molecules, which would result in partition to the bottom phase (red arrows) in our PEG–DEX system. (B) and (C) At increasing SDS concentrations, SDS begins to comingle with, or outcompete, the DOC on the interface of some SWCNT (n,m)s; at sufficient SDS fraction these SWCNTs partition to the top phase (green arrows). The SDS concentration necessary for this for each (n,m) corresponds to the empirical critical point for that (n,m) shown in Fig. 2D. (D) At high SDS concentrations, (almost) all SWCNTs are coated with SDS, or a sufficient fraction of SDS to cause partitioning into the top phase. Note that the number of shown surfactant molecules in each box is not quantitative. However, from left to right the number of unbound DOC molecules remains constant while the number of SDS molecules increases dramatically to reflect equilibrium with the bulk volume beyond the schematic frame. It is possible that the amount and type(s) of polymer(s) affects the competition of the surfactants.

The mechanism driving the phase selection appears to be which surfactant primarily is adsorbed on the specific (n,m) SWCNT interface at any given set of SDS and DOC concentrations. For single surfactant coverage, SWCNTs coated with sufficient DOC partition to the DEX phase in our typical ATPE system, and SWCNTs coated with SDS partition to the PEG phase. A schematic of the mechanism hypothesis is shown in Fig. 7. In the figure, the SWCNTs primarily coated with DOC would have a difference in their chemical potential for insertion into the top *versus* bottom phase, *μ*^0^_T,i_ − *μ*^0^_B,i_, greater than zero, which would lead to a partition coefficient < 1, and thus separation into the bottom phase. It is currently unclear how much comingled adsorption of both surfactants occurs, whether there are changes in fractional surface coverage that lead to direct contributions, rather than surfactant mediated contributions, from the nanotube sidewall, or what the slope of the changeover is from one surfactant to the other with increasing concentration of one component. A significant question is whether defects on the nanotubes broaden the range of this transition, and whether that factor has been accounted for in spectroscopic measurements of the exchange. These are current active areas of research by several groups, as well as a recommended direction for improving ATPE described in a later section.

The primary decision for diameter–structure multistage ATPE at each step of the processing is what concentration of SDS to apply. In Fig. 2C and D a schematic of the separation coefficient functionality for different (n,m) SWCNTs and the empirical conditions, ignoring enantiomer effects, at which each (n,m) reaches *K*_(n,m),i_ = 1 were respectively shown. Using such information, one can choose an SDS concentration that will split some species into the top phase, and some species into the bottom phase, in an ATPE step. For the species denominated in the schematic of Fig. 2C, a good choice for a first ATPE step would be ≈0.72% SDS (at 20 °C, 0.05% DOC) corresponding approximately to the position of the vertical dashed line, which will partition such species as the (9,4), (6,6), and (7,5) into T, the top phase, and (7,6), (8,3) and (6,5) into B, the bottom phase. In the second stage of separation, bottom phase mimic containing 0.05% DOC, but no SDS, is added to T to reduce the total SDS concentration to ≈0.65%, resulting in (9,4) and (8,6) remaining in phase TT, and partitioning of the (6,6) and (7,5) to TB. Similarly, a high concentration SDS mimic top phase is added to B, to raise the overall SDS concentration to ≈0.8%, partitioning (upon mixing and phase separation) the (7,6) and other not shown species into the top phase BT, and leaving the (8,3), (6,5), *etc.* in the bottom phase BB. This process is then repeated with the goal to separate each (n,m) into an ATPE phase by itself, increasing the SDS concentration in a bottom phase, and decreasing it in a top phase to shift the SWCNT partitioning between each stage in the cascade. Alternatively, the DOC concentration can be increased (decreased) for the top (bottom) fraction while maintaining a constant SDS concentration, but in practice this is difficult to implement with fine enough control for generating subpopulations (as the concentration steps would be very small in terms of absolute DOC concentration). It is instead a technique best utilized only to shift all (n,m) species to the opposite phase.

**Fig. 8:**
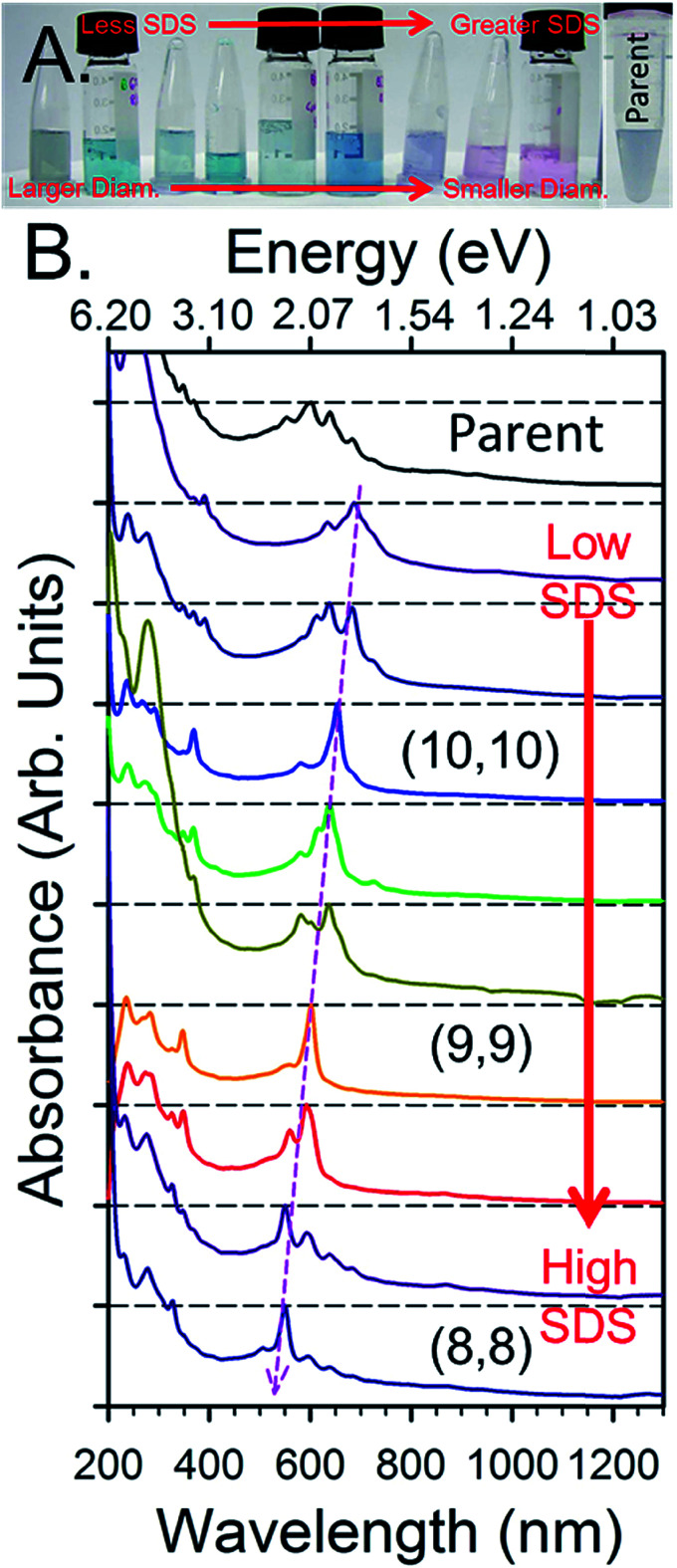
An example of fractions resulting from the gradient process ATPE separation of empty metallic PT SWCNTs adapted from Fagan *et al.*^19^ (A) Photographs of the SDS gradient ATPE separated diameter fractions in order from lowest SDS for extraction (left) to greatest SDS for extraction (right). (B) Absorbance spectra of the fractions SWCNT fractions in order of their appearance in the photograph in panel (A) (left most = penultimate top), the topmost spectrum is of the metallic parent dispersion shown in (A). The changes in the SWCNT species distribution from systematic ATPE separation are demonstrated by the changes in absorbance peak wavelengths, with specifically enriched armchair (n,m) species labelled. Adapted from Fagan *et al.*,^19^*ACS Nano*, 2015, **9**, 5377–5390.

An example of the results from a gradient process ATPE separation on an approximately 1.3 nm average diameter SWCNT population synthesized by the plasma torch (PT) method from Fagan *et al.*^19^ is shown in Fig. 8. Application of the gradient strategy is able to distinguish multiple single (n,m) species fractions despite the many SWCNT species present in the parent population. As discussed in greater detail below, the fractional quantity of each SWCNT species decreases in larger average diameter SWCNTs populations due to the greater number of (n,m)s over which the total mass is distributed. This factor, plus difficulties in judging the success of a partitioning step *via* visual inspection can make separating populations larger than ≈1.2 nm in average diameter challenging, but systematic fractionation will reveal population separation in spectroscopic measurements.

As the multistage process is similar for all ATPE implementations, what differentiates this approach from other variants is that the implicit goal is to collect all (n,m) SWCNT species in different final fractions, rather than a focus on purifying one (n,m) component while disregarding the others. As stated above, both strategies have their place, with multistage (SDS gradient) separations taking more effort and time, but also reducing discarded material. The key benefits to this approach are flexibility in the process to compensate for differences in materials and goals of different laboratories including batch to batch variation in the chemicals used, local laboratory temperatures, the SWCNT source lot, and the desired (n,m) structure to be isolated.

Realistically time is not often available to purify and collect each species separately. In this case a hybrid approach is sometimes used. In this case only a few separation steps are performed in the typical gradient manner, resulting in four to six subpopulations. To all but one of these enough DOC is added to reach a 1% concentration, and only the final subpopulation is purified *via* a further multistage cascade for the target (n,m). If the properties of the starting SWCNT population are consistent with another experiment's, the other populations can be mixed with similar (n,m) distribution samples to increase the absolute SWCNT mass in such fractions before reintroduction to ATPE and additional separation. These concentrated subpopulations then enable isolation of (n,m)s present only in low concentration in the original parent dispersion.

The greatest level of discrimination amongst the SWCNT species is to the level of separating the two enantiomers, (n,m) and (m,n), of the chiral structures. For a number of species including (5,4), (6,5), (7,5), and (10,2), the differences in SDS concentration (for constant DOC concentration) needed to partition the two enantiomers are blatant, on the order of a 10–20% difference. For these species a multistage separation with a relatively small step SDS gradient will resolve two different SDS concentrations at which first one, then the other, enantiomer partitions, as monitored by the presence or absence of the very similar (n,m) and (m,n) optical transitions, from the bottom phase to the top phase. In contrast, for other species the differences are small enough to be unassignable to different enantiomers, or even discriminated at all. As with different (n,m) structures, whether two enantiomers are separated fully in a single ATPE stage will depend on how distinct are their separation conditions and the idealness of the achieved equilibrium. Whether separation of enantiomers is necessary may depend on the purpose for which the sample will be used. The tradeoff between the addition of complexity to the separation (that will be shared by other techniques reliant on the same mechanism) for more specific optical response will need to be evaluated on a case by case basis. Automated multistage gradient sorting is one route to generating better separated fractions, and the other is better identification of the surfactant conditions to perform optimal single stage, or repeated at a constant surfactant concentration, partitioning. Such conditions can be identified through measurements of relative binding affinities of surfactants,^38^ which is an area we highlight as a significant opportunity for improving ATPE in a later section.

#### Setpoint (recipe) ATPE

In what we term “setpoint” ATPE the goal is usually different than the general methodology just described. Introduced by Subbaiyan *et al.*,^26^ a specified, short, set of steps is used to isolate a specific subpopulation, usually a single (n,m). The premise is that the more highly controlled conditions from the short number of steps can be tailored to maximize the discrimination between the target species and all other (n,m) SWCNT species. From the data set in Fig. 2D, or a more recent data set of Defillet *et al.*,^37^ it is clear that most (n,m)s can be reasonably isolated (or significantly enriched in fractional concentration) by selection of the SDS concentration in two ATPE steps while holding constant the DOC concentration. In the first stage a slightly greater SDS concentration than that needed for upper phase partition would be used, followed by a second step at a slight lesser SDS concentration condition to fractionate the desired (n,m) into the bottom phase. However, when using only SDS and DOC it is rare for all of the (n,m) species in a population partition coefficients to be sufficiently distinct to enable high purity isolation in only two steps. As such, “setpoint” ATPE recipes often break the implicit restriction in the general method to changes in only the DOC or SDS concentrations between steps. Other surfactants, salts, or modulating agents can (and perhaps must) be added to maximize the differentiation between the goal and non-target SWCNTs.^27^ The benefits of this approach are that the target species can be rapidly isolated and with less training and difficulty than the general methodology. The drawbacks to this approach are twofold, first a specific recipe for each target (n,m) SWCNT must be developed, and second, that such maximization tends to lead to aggregation and loss to the interface of all other (n,m) species in most published recipes.

#### Other surfactants or trisurfactant + ATPE

Similar to the general method but relaxing the restriction of competing only two surfactant mixtures of SDS and DOC, is the extremely large phase space of competing other surfactant combinations or three plus surfactant mixtures to control the SWCNT partitioning. Even restricting the combination to other bile salt surfactants, or replacement of SDS with sodium dodecyl benzyl sulfonate (SDBS), different partitioning behaviors have been realized in ATPE experiments as well as in gel chromatography experiments.^39^ A significant example for ATPE is the use of SDS–DOC–SC mixtures to isolate (6,5), (7,3) and (6,4) from each other. For SWCNTs of such small diameter the amount of SDS needed to partition the (n,m) structures to the top phases with a fixed DOC percentage both induces an instability of the dispersion against aggregation, and does not particularly well distinguish between the (n,m) structures. Addition of a 3^rd^ component, the SC, alleviates these issues. Although few alternate or multisurfactant competition research efforts have reached publication to date, it is likely that, especially through replacement of the DOC, other partitioning orders will be developed.

#### Acid addition ATPE

A very recently reported variant of SDS–DOC competition driven diameter/structure separation by Li *et al.*^40^ is to use acid addition, rather than volumetric addition or dilution, to change the partitioning of different (n,m) SWCNTs within or between separations. For relatively minor amounts of acid addition to an ATPE system, the authors show that SDS, p*K*_a_ ≈ 1.9, is insensitive to the addition, but DOC, p*K*_a_ ≈ 6.6, can be shifted from a disassociated salt into its acid form. Because deoxycholic acid (DA) is much less soluble than its sodium salt, addition of a concentrated acid to convert some DOC to DA effectively reduces the DOC concentration in solution available for competition with the SDS on the SWCNT sidewall. In the acid addition limit in which sufficient acidification of DOC, and thus its effective sequestration, can be induced with only minor changes to the overall solution environment, acid addition can thus be used to carefully tune the surfactant competition.

Although many diverse modulating chemicals exist for changing the partition of SWCNTs in the ATPE system, the utility of acid addition is specifically notable because it can be accomplished through very small volume additions of concentrated acid, and because it is reasonably reversible through addition of base, with the minor effects of salt concentration increases the main limitation to full reversibility. Using reasonably optimized DOC and SDS concentrations, and adding specified volumes of 0.5 M HCl on the order of 0.1% of the total volume, the authors were able to demonstrate separation of 11 small diameter (n,m) species at purities consistent with Fagan *et al.*^18^ but in significantly fewer steps of ATPE. Reducing the number of steps is important as it results in simpler adoptability of the technique as well as less consumption of polymers and other chemicals. In addition, the authors report successful utilization of 3-surfactant mixtures of SDS, SC and DOC with the acid addition to optimize some separations, with their results implying that the acid addition more weekly affects the SC concentration than the DOC concentration. Whether this results from the larger concentrations of SC used, or the roughly four times greater solubility in water of cholic acid than deoxycholic acid^41^ is unclear, but raises the possibility of differential resolution of SC and DOC adsorbing SWCNT (n,m)s for enabling even finer resolution than purely SDS–DOC competition.

#### ssDNA ATPE

Although this perspective is dedicated to surfactant driven ATPE separation of SWCNTs, most of the technical and practical considerations for single strand (ss)DNA–SWCNT ATPE partitioning are common with surfactant-controlled ATPE. Particularly for DNA sequences that do not result in outlier partitioning of single (n,m) populations, *i.e.*, recognition sequences,^20^ sequential partitioning of SWCNTs with added modulating agent is frequently observed in bench tests. Much like using any new dispersant in ATPE unique orders of separation may be achieved, however, at current knowledge sequences yielding recognition behavior or valuable partitioning behavior are difficult to predict *a priori*. As a separate matter, the use of a DOC to DNA dispersion exchange procedure^42^ to enable subpopulation, *i.e.*, small groups of (n,m)s, enrichment in surfactant ATPE before utilizing an exchange with recognition ssDNA sequences for DNA-ATPE to purify single (n,m)s to extreme purity, or for enantiomeric handedness,^21^ is a strategy that will be discussed in more detail in a later section.

### Considerations and perspectives on automation, scaled production and cost reduction

A significant benefit of the liquid–liquid nature of the ATPE separation method is its likely ability to be successfully scaled to increase mass throughput and to reduce costs. Due to the much higher SWCNT mass loadings at which ATPE can be conducted than gel chromatography or DGU, in our laboratories the initial dispersion and purification by sonication and centrifugation are the rate limiting step. Beyond this barrier, however, there are clear paths for improved scale processing and cost reduction.

For semiconducting SWCNT production, the robust nature of the single step ATPE separation quality from the metallic nanotubes suggest the simple use of larger containers and centrifuges to reach multigram production scales using process flows similar to the batch model outlined above. At larger scales, counter current flow centrifugal contactors,^43^ small models of which process 60+ L h^−1^ flowrates, would allow continual processing (for estimation: 0.5 mg mL^−1^ × 60% yield × 60 L h^−1^ = 18 g h^−1^ semiconducting SWCNTs) for which application markets have not yet developed. Key technologies for cost reduction in either case would include cheap removal of the SWCNTS from the polymer and recovery for recycling of the polymer phases. In addition, reduction of DOC concentration during dispersion, perhaps by utilizing a SC/DOC mixture, while maintaining dispersion quality would greatly help.

For production of individual (n,m) SWCNT species samples, the greatest impediment is the low mass fraction of any given (n,m) in the original synthetic mixture. In small diameter cobalt–molybdenum catalyst (CoMoCat)^44^ or high-pressure CO disproportion (HiPco) synthesis type batches with average diameters < 1 nm to 1.1 nm the relatively small number of (n,m) combinations (≈20 and ≈40 respectively) makes the problem tractable, especially for high abundance species such as the (6,5). The combinatorial problem becomes very significant, however, for even mildly larger average diameter populations as there are more and more (n,m) combinations possible within a fixed range of diameter variation (for DWCNTs the situation is even more challenging).^29^ Fortunately, narrow groupings of (n,m) species are readily separable with only 2 to 3 separation steps, even for larger, >1.5 nm diameter SWCNTs, at bench scale by hand.^19,45^ These sub-populations should then succumb to sequential application of additional ATPE step if the partition coefficients vary by even 5% as in classical separations such as distillation. Unfortunately, these small steps, and difficulty in rapid assessment of progress, are tedious to perform in practice, leaving purification of large diameter species to heroic efforts. Improvements to SWCNT synthesis methods to generate reduced numbers of (n,m) structures in a soot would thus significantly ease isolation of the enhanced content species, and is an active area of research in the SWCNT community, but separations will still likely be required for many species for the foreseeable future.

**Fig. 9:**
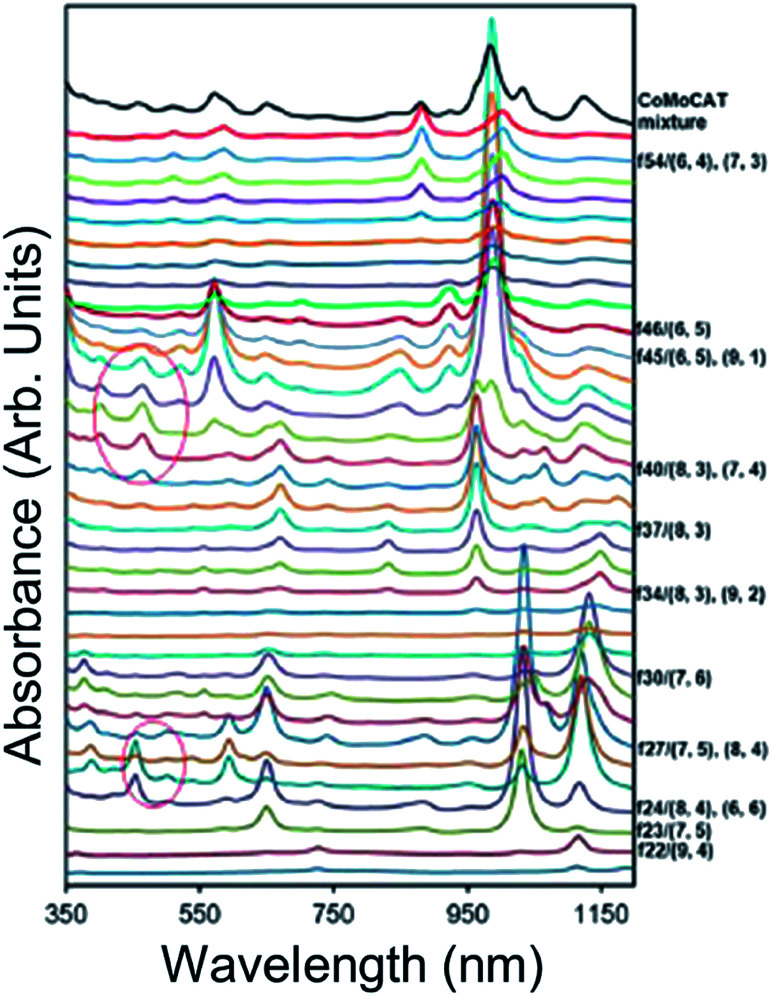
Absorbance spectra of sequential chromatographically-separated SWCNT populations using a CCC instrument. The emergence and disappearance of absorbance features is due to the differential partitioning of the different SWCNT species along an SDS % gradient starting from a low concentration at the bottom of the figure to high concentration near the top. Reprinted with permission from Zhang *et al.*, *Anal. Chem.*, 2014, **86**, 3980–3984. Copyright 2014 American Chemical Society.

Automation of diameter-separation ATPE using a surfactant gradient such as in a counter current chromatography instrument^46,47^ is one possible solution to this problem. An example of the chromatographic output for CoMoCat SWCNTs is reproduced from Zhang *et al.*^46^ in Fig. 9. In those initial experiments dramatic separation of small diameter SWCNTs was achieved with the equivalent of only a few equilibrium single stage fractionations. Minor improvements to both the machine and methodology should enable application of several hundred equivalent equilibrium steps of separation, which would imply baseline resolution of even minor (n,m) components. Acquisition and demonstration of an appropriate machine is of continuing interest.

For either scaled implementation, a simple cost accounting indicates that cost reductions through incorporation of bulk rather than research grade chemicals, and improvements to dispersion, including a reduction in the quantity of DOC used, hold the greatest potential for cost reduction when using an all surfactant strategy.

### Complications ignored in the treatment of the two-phase system as described above

Thermodynamic equilibrium requires that the chemical potential of both phases be equal (if we assume equilibrium is reached), and broadly I have used the assumption above that the two-phase environments are defined primarily by the polymer chemical natures and MWs, with lesser roles played by polymer concentrations (shifting tie-lines) and temperature. I have also described the techniques under the assumption that the total SWCNT concentration, and of any specific (n,m), is too low to affect partitioning of the either the polymers or other components (including other SWCNTs) due to thermodynamic effects. This second is likely to be a good assumption from an equilibrium standpoint, as even 1 mg mL^−1^ SWCNTs is ≈0.1% mass concentration. However, such concentrations can be problematic due to mass transfer effects, in particular interfacial trapping, and reduced yields (if surfactant concentrations that induce or allow aggregation are encountered). The other assumption, namely that of little to no effect of each additive's concentration to the polymer phase equilibrium, is less viable for the addition of the surfactants and other modulators, such as salts, redox agents, and acids or bases. Heuristically, in most instances these additives do not appear to strongly affect the partitioning of the other additive components, *i.e.*, the partition coefficient of each surfactant remains independent, but instead primarily affects factors such as the precise compositions of the polymers at the phase boundary as was shown in Fig. 1. However, measurements to support these assertions are limited, and greater understanding of the effects of additives on the partitioning of other non-SWCNT components could be useful. Some observations that do imply intercomponent interactions are that increases in the SDS content tend to shift the polymer phase coexistence curve towards greater mass fractions of both polymers, as in the shift of the surfactant free and with surfactant curves in the Fig. 1, and that DOC concentrations above ≈0.2% greatly increase the rate of two-phase separation. The latter implies an increase in the difference in chemical environment, as described by hydrophilicity–hydrophobicity, of the two-phases. Of course, SWCNTs are usually all in the dextran phase at such DOC % compositions.

Another complication ignored above is the impact of SWCNT length on the separation. With regards to separability of SWCNTs of various lengths, it has been previously noted that the surfactant concentrations for which *μ*^0^_T,i_ − *μ*^0^_B,i_ = 0, and thus the partition coefficient is 1, are independent of the SWCNT length (neglecting end effects).^6^ SWCNT length, which might be expected to linearly amplify a per unit length chemical potential difference, may thus change the slope of the partition coefficient functionality, but will not affect the partition order of (n,m) species. The use of length separated populations for ATPE is thus beneficial, because a narrow length distribution will therefore yield a consistent and sharper slope to *K*_(n,m)_ (SDS, DOC), but is not necessary.

Alternatively, as noted in Khripin *et al.*,^16^ each ATPE step could be used for, or lead to, length separation of a polydisperse SWCNT length population if *μ*^0^_T,i_ and *μ*^0^_B,i_ are proportional to the SWCNT surface area. In practice however, we rarely observe length distribution changes from our application of ATPE, or at least not to the potential for concern suggested by a linear scaling of the chemical potential difference. Evidence for this assertion comes from analytical ultracentrifugation results determining essentially unchanged length distributions of parent and ATPE sorted (n,m) enriched fractions.^48^ Several reasons for this empirical observation could be artifactual. Most likely our dispersion and pre-ATPE processing simply result in a length distribution narrow enough to not affect the partitioning mass balance within the resolution of surfactant concentration steps we specify during ATPE. Alternatively transport phenomenon might provide a counteracting bias to length selection from equilibrium thermodynamics. Another potential hypothesis is that the separation mechanism, especially for SDS–DOC sorting, results in a significant change of the chemical potential difference for each (n,m) SWCNT species at the critical concentration at which one surfactant replaces the other. This hypothesis would imply that all lengths of SWCNTs should be separable by ATPE at both similar surfactant concentrations and with sharp *K*_(n,m)_ slopes. This is a testable hypothesis through the surfactant competition experiments suggested in the next section.

### ATPE development opportunities

For all of the above discussion, ATPE of SWCNTs is still a developing field with significant opportunities for resolving both fundamental and applied research questions. Several of those questions, but by no means a full and complete list, are discussed below.

#### Can the functionality of partition coefficients for specific (n,m)s be rapidly evaluated with spectroscopic observations, advanced fitting software, and simple temperature and global composition control?

If the hypothesis that the polymers are inactive in the ATPE separation of the SWCNTs except as acting an analyzer for which surfactant dominates the interface of each (n,m) is true, then it follows that conducting an ATPE separation at any given set of surfactant concentrations is actually unnecessary for evaluating which SWCNT (n,m)s will partition to top and bottom phases at those conditions. Conceptually then, if the surfactant coverage can be determined for a given (n,m) in the absence of the polymers *via* other methods, then we should be able to determine the surfactant concentration combinations that will result in effective ATPE partitioning outside of the two-phase system. Even if a polymer(s) play a role in modulating the surfactant competition for the SWCNT interface, as long as only one is important this approach should work.

In the case of SDS and DOC dispersions of SWCNTs, the wavelengths of each individual (n,m)'s optical transition are different due to solvatochromic effects. Because of this, the dominating adsorbate at any pair of SDS and DOC concentrations should be discernable directly from absorbance or fluorescence spectroscopy in the absence of the polymer. Such a strategy has been significantly used to investigate the replacement of DNA with various surfactants,^49,50^ but only in a more limited fashion for the competition of different surfactants. Essentially the surfactant concentration conditions at which the SWCNT optical properties shift from being reflective of coverage by one surfactant to the other would be identified as likely very similar to the partition coefficient = 1 point.

A primary reason for this is that in as-synthesized (n,m) population samples spectral observations are complicated by spectral congestion of many overlapping transitions from different (n,m)s and their enantiomers, as well as contributions from damaged or impure SWCNT populations. However, these complications will be dramatically reduced for single (or few) (n,m) separated fractions, and could be definitive, even in the absence of (n,m) from (m,n) resolution, of critical concentrations at which one surfactant is replaced by the other. Such measurements tracking the (different) peak positions for coverage by one surfactant *versus* the other *via* absorbance or fluorescence spectroscopy of few SWCNT populations have been reported against flavin mononucleotide (FMN) dispersants,^38,51^ but not systematically for small molecule surfactant pairs. Competition of DOC and SDS or other surfactant pair combinations on well sorted (n,m) populations should enable rapid screening of conditions that may lead to novel separation capabilities.

Such experiments may also realize answers to the questions raised above on the effects of defects on the partitioning, and the potential for direct nanotube sidewall contributions to the phase affinities. Another interesting experimental test beyond ATPE for the shifting surfactant coverages could be density gradient ultracentrifugation, as the change in surfactant layers will likely result in different buoyant densities of the dispersed SWCNT.^52^ Such results may thus enable fuller identification of separation conditions and predictability for separations such as those performed by Ghosh *et al.*^8^

#### What is the timescale of the surfactant coating competition on the SWCNT surfaces. Does the exchange happen entirely before ATPE phase separation, or is it partial (biased) during and completes later?

An fascinating follow-up question to probe the spectral determination of surfactant coverage just described is whether the same surfactant coverage that selects for partitioning is the same surfactant coverage that exists on the SWCNT surface after ATPE phase separation. This information would affect how the hypothesized mechanism for phase selection acts. Comparison of results in the absence of polymers, and after ATPE separation such as in Subbaiyan *et al.*^26^ could resolve this question.

#### Are SDS, DOC, SC, the best set of surfactants for ATPE sorting of SWCNTs?

Facilitated screening of surfactant competition for (n,m) surfaces would also ease the investigation of other surfactant molecules and combinations without the mass requirements of performing the two-phase separation. While many other surfactants have been tested, if not all reported, a consistent set of spectroscopic variation studies, such as in Park *et al.*,^38^ might enable identification of conditions not tested in limited ATPE separations. Amongst these, while multiple bile salt molecules have been tested for ATPE, several commercially available ones such as muricholic varietals, which albeit are currently much more expensive, have not yet been reported on for utility in SWCNT ATPE separations. Design, tailoring or identification of other effective surfactants for competition could also yield dramatic improvements.

#### Are we using the optimal mixture of PEG and dextran MW mixes?

Recently we discovered for DNA-ATPE separations that the degree of interfacial trapping could be drastically reduced and additional DNA sequences utilized for SWCNT separation with polymer:polymer combinations differing from the 6 kDa PEG and dextran 70 that have been the basis of most SWCNT ATPE. The work by Lyu *et al.*^53^ found instead that combinations of a lesser MW PEG with a greater MW dextran, approaching optimization at 1.5 kDa PEG and 250 kDa dextran, performed better in both reduced interfacial trapping and in the number of usable sequences. Although in our prior work with surfactant ATPE we did not observe strong dependencies with MW of the polymers such that significant optimization was not pursued, this newly informed variation may be a vector for improving the ATPE process.

#### How specific can we make really large diameter SWCNT separation?

In Fagan *et al.*^19^ it was shown that an increase in the DOC concentration enabled greater resolution in SWCNT diameter, for feasible SDS concentration control, when separating larger diameter synthesis SWCNTs such as electric arc SWCNTs. Furthermore, through an analysis of the differences in SDS concentration used to isolate specific (n,m) clusters it was extrapolated that the limit at which (n,m) resolution by SDS–DOC competition would no longer work was >1.7 nm. However, although resolution of >1.8 nm diameter subpopulations has recently been shown,^45^ no single chirality SWCNT population larger than ≈1.5 nm in diameter has yet been demonstrated. In the absence of automated multistage extraction, such as by a CCC, and the difficulty in extracting single species (due to the low concentration of each (n,m), enantiomer effects with DOC, challenges in visual (or even spectrometer) assessment, and broadened optical transitions for water-filled SWCNTs), no literature contributions have yet reported fractions either. Similar difficulties in resolving (n,m) species are also encountered even with heroic efforts in ultracentrifugation separations.^54^ The author can happily report forthcoming progress on this front through the use of 3-surfactant competition strategies and fractionation of concentrated narrowed sub-populations, but much work remains.

#### How can we best use surfactant ATPE with DNA exchange to drive technology development?

There are several obvious pathways in which to combine surfactant ATPE with ssDNA exchange technology to generate more pure (n,m) SWCNT samples, to probe the binding of DNA on SWCNT surface, or to drive technology development. For the first, the abilities of ssDNA enabled ATPE are somewhat different from those of surfactant ATPE, and can highly purify enantiomers of some (n,m)s in a short number of steps.^21^ It is a clear strategy to limit DNA consumption through first using surfactant ATPE to make rough cuts in a SWCNT (n,m) distribution before exchange and final purification. Larger scale separations, or the isolation of empty or alkane-filled SWCNTs *via* surfactant methods, which are substantially easier than with DNA dispersions, for exchange and purification may enable better determination of intrinsic properties and new applications. In the second case, multiple prongs of analysis, for instance by using analytical ultracentrifugation to probe structural differences as a function of exchanged ssDNA sequence, or amplification in an aptamer selection strategy to identify strong binding DNA sequences, would be of high interest. Lastly, the narrowing of SWCNT properties through structure sorting by surfactant ATPE could enable sensing applications such as the electronic nose or molecular perceptron^55^ through systematic and repeatable generation of elements with differential responses to stimuli.^56,57^ Each of these ideas, and likely similar research directions are paths I believe will yield exciting results.

### Technical and practical considerations for surfactant-SWCNT ATPE at the bench scale

Over the last several years I have hosted many visitors on both short and long visits for demonstration and dissemination of the ATPE methods described above, as well as heard from many other research groups with questions when trying to implement the technology. Separation *via* ATPE of SWCNTs in expansion or replication of the work initiated at NIST has now been reported across multiple groups on several continents. To aid in even broader adoption of the technology there are many technical and practical considerations when implementing ATPE that also deserve discussion and that may aid in the development of better future separations. These considerations are presented below.

#### SWCNT quality and quantity

In my experience, several factors outside of the ATPE process are frequently the source of difficulty in replication and utilization of the ATPE separation method. The first of these is the method and conditions of dispersion, usually by sonication. For instance, dispersion in any of several common surfactants, such as SDS, only supports a limited concentration of fully individualized SWCNTs,^59,60^ for SDS on the order of 100 μg mL^−1^, before spontaneous bundling start to occur. This can be fine for spectroscopic studies, but any bundles cannot be separated in the ATPE system, and a low mass concentration input practically ensures dilute end samples.

In my preference, and by most metrics reported in the literature, ssDNA^58^ and DOC^59,60^ are the best available dispersants for mass yield of individualized SWCNTs *via* sonication in an aqueous environment. For DOC, Blanch *et al.*^61^ determined that peak SWCNT dispersion yields occurred for DOC concentrations between 1% and 2%. We have also found 45 min to 1 h of tip sonication at a nominal power of ≈0.9 W mL^−1^ to a cooled vessel to provide a reasonable optimization of conditions. Mass loading of SWCNTs is typically 1 mg mL^−1^, with yields, as monitored by fraction of absorbance retained, after 2 h of ≈38 000*G*, *G* = 9.81 m s^−2^, in a rotor of *k*-factor^62^ = 769 (see Methods in the ESI†) on the order of 40–50% of the (non-SWCNT peak feature) absorbance for most SWCNT materials, although this can be up to ≈95% for some small diameter synthesis populations. As a reference, although the absolute concentration reachable in a SWCNT dispersion depends on the SWCNT length distribution, the maximum concentration while avoiding formation of separated SWCNT phases is ≈2 mg mL^−1^. Easily handled SWCNTs concentrations are typically below 1 mg mL^−1^. These parameters should not be viewed as prescriptive, but are offered as reference points that have yielded good results, and I note that even after almost 20 years, the evaluation of initial dispersion quality is an ongoing research topic.^63,64^ In fact, transferable optimization of dispersion, or research enabling equivalent dispersion yield and quality with less time or using a mixture of SC and DOC to reduce the absolute initial DOC content would be of great value for decreasing costs and improving mass throughput of ATPE separations.

A second factor that at least eases the use of ATPE for separations, and that may be a requirement for achieving high purity fractions with the largest diameter SWCNTs, is the use of alkane-filled SWCNTs^65,66^ or empty SWCNTs, *i.e.*, SWCNTs with closed endcaps,^67,68^ rather than water-filled SWCNT populations. Such nanotubes have significantly narrower optical transitions than water-filled nanotubes, easing visual assessment of separations, and by virtue of only having one water-exposed surface, likely experience a less heterogeneous chemical environment in the aqueous dispersion. Note that the mass fractions of closed ended, *i.e.* potentially dispersed as empty, open-ended but pristine, and open ended and notably oxidized SWCNTs, which will have decreased optical feature size, in a SWCNT source depends on the details of both the synthesis method and any manufacturer applied purification. In our labs we also generally purify empty, water-filled and alkane-filled SWCNTs from remaining small bundles, slowly sedimenting impurities, and morphologically-defective SWCNTs *via* a rate-zonal centrifugation.^69^ This process does add a significant amount of effort, and results in loss of some valuable material, but also generally dramatically improves the SWCNT population purity and optical absorbance spectrum. Whether the fractional sample mass primarily eliminated in this step is mostly an intrinsic property of the SWCNT production method and soot morphology, or mainly due to non-optimal dispersion processing, is unknown, and an area of ongoing interest.

#### Taking advantage of temperature

As discussed in the section describing the improved semiconducting–metallic SWCNT separation workflow in Fig. 4C, temperature is known to be a powerful parameter for affecting ATPE separations.^25^ There are two primary manners in which the temperature can affect the separation. One is through changes in the SWCNT partition, as lower temperatures generally bias partitioning into the bottom phase, and the second is through affecting the compositions of the phase boundary. In a cooled, but non-uniformly mixed, vial sometimes both effects can be seen at once, with distinct formation of three or even four layers after centrifugation.

**Fig. 10:**
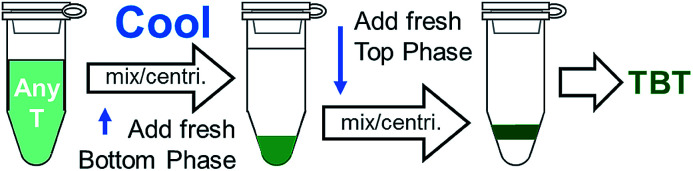
Schematic of the workflow to concentrate a dilute top phase fraction using a temperature swing. Cooling to <10 °C, and the addition of a small amount of bottom phase, results in partitioning of all SWCNTs to the bottom phase. Returning to room temperature, and adding a small volume of top phase, re-enables partitioning into the top phase. Large volumetric concentration can be achieved with minimal SWCNT mass loss *via* this method.

Both of the two effects are readily utilized to transfer dilute top phase fractions to the bottom phase through use of a chiller bath. In many cases, such as in the alternate semiconducting–metallic separation workflow given in Fig. 4B, it can be useful to transfer these fractions to a new bottom phase, but without significantly adjusting the surfactant concentrations. Frequently we finish top phase fractions by first chilling them to <10 °C with an addition of 5% to 10% bottom phase mimic to concentrate them in the bottom phase, and then rewarming to room temperature and adding ≈1/4 to 1/3^rd^ volume of top phase mimic with a high SDS content. Overall the process thus results in an ≈40× concentration increase of the sample in the final PEG phase with minimal SWCNT mass loss. Concentration of dispersed particles by repartitioning is not a new development,^22^ but is elegantly accomplished through the temperature swing for SWCNTs. A schematic of this process is shown as Fig. 10.

Separately, minor chilling or heating can be used to shift the partition coefficient curves mildly to either transfer more of a desired species into a top phase, or to transfer an undesired species out. Because the partitioning is so sensitive, it is useful to monitor the lab temperature during the course of a multistage ATPE experiment. Normal temperature in our labs ranges from 19 °C to 22 °C depending on the room. Temperatures above 24 °C also are heuristically found to result in poor fidelity in separations, and an increased likelihood of aggregation and should be avoided. Heating may also result in the polymer:polymer phase coexistence boundary shifting such that the composition being utilized is in the single phase region, and thus no separation will occur.

#### Nucleation *versus* spinodal decomposition for phase separation

Avoid overall polymer compositions close to the phase coexistence curve when adding mimic phases. For ATPE separations it is highly desirable to work at polymer concentrations in the spinodal decomposition window of the phase diagram rather than to rely on nucleation.^22^ The two primary reasons are the much shorter length scale of the chemical gradient, on the order of microns with spinodal decomposition, and domination of the polymer two-phase separation kinetics by the coalescence step, rather than by the nucleation rate. A good rule of thumb is that the minor phase should be at least 10–15% of the total volume.

Typically, nucleation will otherwise be encountered either when a separated top or bottom phase cools, as temperature reduction shifts the two-phase coexistence line such that the equilibrium content of the minor component is reduced (for PEG–DEX near room temperature), or when purposely chilling a top-phase fraction to concentrate a sample *via* repartitioning. In the first case addition of a small amount of water will prevent nucleation, and in the second case addition of sufficient primary bottom phase polymer to reach the spinodal window is advised (enough to raise the global mass concentration ≈ 1% is typically more than sufficient).

#### Composition of mimic phases

Except in some special cases, for surfactant ATPE we generate the so-called mimic phases at compositions approximating the phase boundary points, but without including the minor component polymer. Calculating and plotting the effects of such a choice on the polymer:polymer phase diagram in Fig. 1, it is clear both that the resulting phase compositions are not precisely on the same tie-line, but also that the degree of drift from the tie-line is minimal even over multistage separation. The reasons behind this choice are two-fold; one, it is much faster and easier to generate additional mimic phase volume without worrying about having to centrifuge and separate the resulting layers, and two, that in our lab mimic phases including both components often results in bacterial colonization and poor shelf stability of the mimic phases. Additionally, if the temperature of the lab drops, such as during overnight hours, at any time after formation a true mimic phase may reseparate due to the shifted two-phase boundary.

#### Ending in the PEG-rich phase

An important consideration for downstream processing of produced, fractionated, samples is which phase to end a separation in. In almost all cases it is preferable to end in the upper, PEG-rich, phase in the PEG–DEX ATPE system primarily discussed in this contribution. The two primary reasons for this are; one, that the 6 kDa PEG is much faster and easier to remove by filtration or dialysis than the ≈70 kDa DEX (and especially its high MW tail), and two, as in mixed PEG–DEX mimic phases, the DEX is an acceptable growth medium for bacteria (at least in our labs). Always ending in a single phase also provides the advantage of needing a single reference phase for spectroscopy. Typically, in our labs, final top phase fractions are diluted by equal volumes of 2% DOC in water solution to establish a reasonably uniform surfactant environment across all samples, and one that is stable for long term storage.^60^ If acid addition was used to modulate partitioning neutralization is also appropriate at this time. If ultrafiltration cells are used to concentrate a PEG-rich top phase diluted with DOC the mass yields are typically near 100%, and by sequential dilution and concentration, the PEG content can be reduced rapidly to negligible levels.

#### PEG precipitation exchange

Alternatively, and especially for dilute, large volume samples, it is possible to utilize the depletion force provided by a concentrated PEG solution (*i.e.*, the final top phase concentration) to precipitate the SWCNTs into a small volume pellet for later dilution.^70^ In this case addition of DOC to a 1% final concentration from a stock solution such as 80 g L^−1^ DOC in water is recommended, as well as addition of some salt and refrigeration. Lesser PEG concentrations will precipitate SWCNTs on the basis of their length, but at 8–10% PEG all of the SWCNTs will be precipitated. SWCNT mass loss is generally negligible with this method.

#### Aeration during mixing

We have observed that significant aeration of samples during the mixing step of surfactant ATPE is correlated with increased aggregation and trapping of SWCNTs at the interface. Swirling to mix samples, rather than shaking or vortexing, reduces the observed amount of interfacial trapping. It is unclear whether surfactant adsorption to the created air–water interfacial area, or interactions between confined SWCNTs in the thin regions of draining fluid between the generated bubbles are to blame, but either scenario is easily avoided.

#### Centrifuge rotor and vial geometries

Similar to aeration, use of particularly narrow vials, with aspect ratios > ≈5, and fixed angle centrifuge rotors are also observed to increased aggregation. For the fixed angle centrifuge rotor, this is likely from the impact into and accumulation along the vial wall of the droplets during phase separation. Such a band can oftentimes be observed distinct in position from the mobile meniscus after centrifugation in such systems. For primary separations we recommend use of swinging bucket centrifuge rotors if available. At small scales, or for resolving the meniscus from a larger volume separation, a small volume fixed angle rotor can still be a valuable tool and are used for such purposes in our labs. For example, this would explicitly entail extracting the small volume of liquid that includes the meniscus and some of one or both phases, pipetting into the small volume centrifuge tube, remixing or not depending on one's goal, and centrifuging to clarify the two phases to a greater degree than practicable in the large volume tube.

#### Other equipment

Due to the practical independence of the ATPE partitioning with SWCNT concentration, it is preferable to work with SWCNT concentrations as large as are practicable. In less purified dispersions, or when nearing surfactant concentration conditions that can lead to aggregation, the working SWCNT concentration should be lower, but in many steps the practical limit is that at which the interface can be visualized in the short path length of the pipette tip (for hand separation). Use of equipment such as stirred ultrafiltration cells, or implementing concentrating steps, *e.g.*, a temperature swing to induce phase shifts, are encouraged.

Another easily implemented methodology is the use of 1 mm pathlength cells for absorbance measurements. Except for extremely dilute samples, many absorbance spectrophotometers have very high precision near zero absorbance, and it can be particularly valuable to measure samples produced in ATPE in short pathlength cuvettes. In particular, in a 1 mm quartz cuvette, absorbance from water in the NIR between 700 nm and 1880 nm reaches a maximum value of only ≈1.4, well within the linear range of most instruments. This means that, with using a good reference spectrum, SWCNT absorbance features can be measured across this range without the need for D_2_O, and enabling spectroscopic access to optical transitions of (n,m)s that would otherwise be obscured by the absorbance of H_2_O. Additionally, the absolute contribution of scattering to the apparent absorbance is a function of pathlength, and so otherwise occluded short wavelength regions can also be accessible.

#### Measurement issues

Beyond the use of short pathlength cuvettes for absorbance measurements there are other measurement issues with assessing success of ATPE, or any other, SWCNT separation method. Briefly these include issues such as solvent absorbance and other in-filter effects in fluorescence measurements, the vast variation in radial breathing mode (RBM) Raman cross sections with (n,m) and excitation wavelength,^71^ and a scarcity of spectroscopic instruments, particularly fluorimeters, that operate in the 1600 nm to 3000 nm window desirable to probe larger diameter nanotubes. These issues are addressed in detail elsewhere, and can be minimized both through good experimental technique, developments in fitting software,^72^ and published tabulated properties by researchers^73,74^ in the SWCNT field.

## Conclusions

This capabilities of ATPE for SWCNT separations are powerful and multifaceted but can be confusing for the new practitioner. In this review I have sought to clarify differences between alternate separation strategies, elucidate reasons behind process decisions, and provide a foundation for better separations. In addition, the look forward identifies clear routes for both fundamental and applied research to improve the technique and to realize scaled utilization. I hope that these perspectives will advance the field and enable new applications from the ATPE method.

## Funding statement

This effort was funded through internal National Institute of Standard and Technology funding.

## Conflicts of interest

There are no conflicts of interest to declare.

## Supplementary Material

NA-001-C9NA00280D-s001
