# Supplementary material for: Aqueous two-polymer phase extraction of single-wall carbon nanotubes using surfactants
Source: Nanoscale Adv. 2019 Jul 11;1(9):3307–24. doi: 10.1039/c9na00280d (PMC9417344; doi:10.1039/c9na00280d)
Supplement: NA-001-C9NA00280D-s001 [file NA-001-C9NA00280D-s001.pdf]

Electronic Supplementary Material (ESI) for Nanoscale Advances.
This journal is © The Royal Society of Chemistry 2019

Electronic Supporting Information (ESI) for

Aqueous Two-Polymer Phase Extraction of Single-Wall Carbon Nanotubes using Surfactants

Jeffrey A. Fagan*

Materials Science and Engineering Division
National Institute of Standards and Technology, Gaithersburg, MD USA 20899

*Corresponding Author: Jeffrey.fagan@nist.gov

Official contribution of the National Institute of Standards and Technology
Not subject to copyright in the United States

This supplemental information contains workflows for various diameter populations of single-wall carbon nanotubes (SWCNT)s.

Certain equipment, instruments or materials are identified in this paper in order to adequately specify the experimental details. Such identification does not imply recommendation by the National Institute of Standards and Technology (NIST) nor does it imply the materials are necessarily the best available for the purpose. Where provided, uncertainty is reported as one standard deviation.

Outline

1. Acronyms
2. Methods Information (SWCNT Preparation)
3. Description of Mimic Phases used in this Supplemental
4. Workflow Schematics for Various Diameter SWCNT Populations
    A. Cobalt-Molybdenum Catalyst (CoMoCat) Synthesis
    B. High-Pressure CO Disproportionation (HiPco) Synthesis (and similar)
    C. Plasma Torch (PT) Synthesis or Laser Vaporization (LV) Synthesis
    D. Electric Arc (EA) Synthesis
    E. Floating Catalytic Decomposition (FCCD) Synthesis
5. Multistage Data Record Sheet Example

Part 1. Acronyms

aqueous two-phase extraction (ATPE).[1]

polyethylene glycol (PEG)

dextran (DEX)

sodium cholate (SC)

sodium deoxycholate (DOC)

sodium dodecyl sulfate (SDS)

Sodium hypochlorite (NaClO)

Semiconducting-Metallic separation (S/M)

SDS-DOC ATPE separated top phase (T)

SDS-DOC ATPE separated bottom phase (B)

SDS-SC ATPE separated top phase (M_T)

SDS-SC ATPE separated bottom phase (M_B)

Part 2. Methods Information

SWCNT powders from which previously unpublished results are shown or described were as-produced and purified electric arc synthesized SWCNT powders, lots AP-A218 and P2-02-A011, from Carbon Solutions of Riverside CA. Sodium deoxycholate (DOC, BioXtra >98 %, Sigma), sodium cholate hydrate (SC, BioXtra >99 %, Sigma), sodium dodecyl sulfate (SDS, BioXtra 99 %, Sigma), eicosane ($C_{20}H_{42}$, 99%, Aldrich), iodixanol (sold as Opti-Prep, 60 % w/v solution, Sigma), deuterium oxide (D_2O, 99.8 %, Cambridge Isotopes), dextran 40, dextran 70, dextran 250 (TCI, Tokyo, Japan), polyethylene glycol PEG (6 kDa, Alfa Aesar) were purchased and used as received.

Dispersions of SWCNT populations, and either rate-zonal purification or separation of empty from water-filled SWCNTs, were performed as previously reported. Briefly, dispersion of as-received, purified or alkane-filled, SWCNT soot was *via* tip sonication (generally 45 min, ≈ 0.9 W/mL), with the vial in an ice-water bath, at a nominal concentration of 1 mg/mL of SWCNTs in 20 g/L DOC solution. Initial purification to remove large aggregates and non-nanotube components was performed *via* centrifugation in a J2-21 high-speed centrifuge (JA-20 rotor, 1885 rad/s (18 kRPM), 2 h) and collecting the resulting supernatant. This sonicated-centrifuged dispersion is subsequently layered (≈ 8.6 mL) above a dense race layer (28 mL) comprised of either 10 % (w/v) or 12 % (w/v) iodixanol and 10.0 g/L DOC in 36.2 mL OptiSeal tubes for rate-zonal purification (VTi 50 rotor, 5236 rad/s (50 kRPM), 2 h 45 min, 20 °C) in a Beckman L80-XP ultracentrifuge to remove small bundles and reduce the content of morphologically impure SWCNTs.

Stirred ultrafiltration cells (Millipore) were used to concentrate SWCNT dispersions, reduce iodixanol or ATPE polymer concentrations to negligible (<< 1 ug/mL) levels, and to reset the DOC concentration to 10.0 g/L before ATPE, and for finished samples. Different sizes of the ultrafiltration cells from 76 mm to < 25 mm diameter (the membrane area) were utilized depending on the volume of sample. Regenerated cellulose membranes of 30 kDa or 100 kDa pore size were primarily utilized.

Separation of the semiconducting SWCNTs from the metallic SWCNTs in each population was achieved *via* ATPE[1] using the redox adjustment advance of Gui *et al.*[3] as described in workflow of Figure 4B or 4C, as described in the main text.

Absorbance spectroscopy was performed on a Cary 5000 UV-visible-near infrared (UV-vis-NIR) absorbance spectrometer through 1 mm path length cuvettes at room temperature. Samples and reference solutions were measured in separate runs and the reference spectra subtracted during data analysis. Measurements were conducted within subranges of 185 nm to 2500 nm depending on the SWCNT sample, but always with a 1 nm step, a 2 nm bandpass, and an integration time of 0.1 s/step.

Presented ESI work flows are additionally based on results from a number of SWCNT sources including: lots AP-A218 and P2-02-A011, from Carbon Solutions of Riverside CA; CoMoCat SG65i SWCNT powder, lot SG65i-L55, from Chasm Nanotechnologies, Norman, OK; as-produced SWCNT powder, lot May-18, from NoPo Technologies of Bangalore, India; Tuball SWCNT powder, lot 4-1803 2014 from OCSiAl, Russia.

Some SWCNT dispersions were made with SWCNT populations comprised of alkane-filled SWCNTs.[2] Briefly, to generate an alkane-filled population for larger diameter SWCNTs, the alkane and an open-ended nanotube soot are first incubated together above the melting point of the alkane for >12 h. During this time the alkane is ingested into the endohedral volume of the nanotubes, which have open ends from their purification process, resulting in filling of the entire population of nanotubes with the alkane. After incubation, the alkane@SWCNT powder was rinsed with heptane, filtered against a membrane (VVLP, 0.1 μm, Millipore), rinsed with heptane against the membrane, and allowed to fully dry at room temperature.

Part 3. Description of Mimic Phases used in this Supplemental

Except in some special cases, for surfactant ATPE we generate the so-called mimic phases at compositions approximating the phase boundary points, but without including the minor component polymer. Calculating and plotting the effects of such a choice on the polymer:polymer phase diagram in Fig. 1 in the main text, it is clear both that the resulting phase compositions are not precisely on the same tie-line, but also that the degree of drift from the tie-line is minimal even over multistage separation. The reasons behind this choice are discussed in the main text.

To simplify recording of mimic phase compositions a shortened notation is used. Written in brackets, we report, in order [C_{DEX}, C_{PEG}, C_{DOC}, C_{SDS}, C_{SC}], in which C_i is the concentration of the denoted component. An example of the type of worksheet we use to rapidly record concentrations, volumes, temperature and fraction numbers is included at the end of this document. An electronic version as a separate, editable, file is available from the author on request.

Common Stock Solutions we use are:
Polymers: (25 and 40) % (mass/mass) PEG; 20 % (mass/mass) DEX
Surfactants: (1, 2, or 4) % (mass/vol) DOC; (4.5 and 10) % (mass/vol) SC; (4.5 and 10) % (mass/vol) SDS
Oxidant: 1/100th dilution with 18 MΩ water of (10-15) % chlorine content NaClO (value reported by manufacturer, new dilution of oxidant prepared fresh daily)
Stock solutions of polymers of greater concentration exhibit increased viscosity that makes accurate pipetting difficult. Stock solutions of SDS and SC of 5 % would also work, and allow for easy dilutions, 4.5 % is used in our labs for historical reasons, and use is continued because formation of 45 mL volumes of mimic phases is straightforward.

Common Mimic phase used are ([C_{DEX}, C_{PEG}, C_{DOC}, C_{SDS}, C_{SC}]) in nominal percent concentration:

a. Semiconducting-Metallic Separation[3]
    a. top phase [0, 11.1, 0.0, 0.7, 0.9]   note, 11.1 % is 20 mL of 25 % PEG + 25 mL other components
    b. bottom phase [0, 15, 0, 0.7, 0.9]

    note, for separating (6,5), (6,4) and (7,3) to the top phase [0, 11.1, 0.0, 1.15, 0.9] is used.
b. Diameter-Structure Separation[4,5]
    a. Small diameter nanotube populations top phase [0, 11.1, 0.04, 1.2, 0]
    b. Small diameter nanotube populations bottom phase [0, 15, 0.05, 0, 0]
    c. Large diameter nanotube populations top phase [0, 11.1, 0.1*X*, 0.7, 0]
    d. Large diameter nanotube populations bottom phase [15, 0, 0.*1X*, 0, 0]

        X depends on the nanotube diameter.

Part 4. Workflow Schematics for Various Diameter SWCNT Populations

All SWCNT populations start at 10 g/L (nominally 1 %) DOC concentration in water. To reach separation conditions for ATPE the DOC concentration must be reduced, although to varying degrees depending on the nanotube type.

Note that within the workflow schematics the concentrations specified [% DOC,% SDS, % SC], are global concentrations. Arrows are intended to represent approximately one stage of separation, although generally the fractions labelled with specific (n,m) species may need more processing steps to reach very high purities. Where they occur, asterisks are used to save space when specifying the separation occurs at the "*" specified concentration.

Preconcentration:

For each of the following workflows, one or two "preconcentration" steps are applied before the first separation step.

2 parts 20 % DEX + 2 parts SWCNT dispersion + 1 part 40 % PEG. $\rightarrow$ [8, 8, 0.4, 0, 0] global composition.

All SWCNTs will partition to the bottom phase. Top is discarded.

1 part Bottom Phase (PC #1) + 1 part 12.5 % PEG $\rightarrow$ 0.2 % DOC, while staying $\approx$ on the tie-line

All SWCNTs will partition to the bottom phase. Top is discarded. Move onto separations.

Remember to end separations in the PEG-rich Phase!

In almost all cases it is preferable to end in the upper, PEG-rich, phase in the PEG-DEX ATPE system. If your final sample is in a bottom phase, do one more ATPE step with a reduced DOC % and/or increased SDS % to shift the sample into the upper phase (the sample can often be successfully concentrated by partitioning into a smaller upper phase volume in this step as well). More comments are in the main text.

A. Cobalt Molybdenum Catalyst (CoMoCat) SWCNTs

CoMoCat synthesis method SWCNTs, the most common of which are SG65i type, typically includes a narrow distribution of SWCNTs ≈ 0.7 nm to 0.85 nm (carbon center to carbon center diameter definition). The SG65i population is strongly biased to the (6,5) structure for semiconducting (n,m)s, and in our experience to the (7,4) for the metallic population.

<table>
<tr><th>Normal Processing (Gradient Strategy)</th><th></th></tr>
<tr><td>
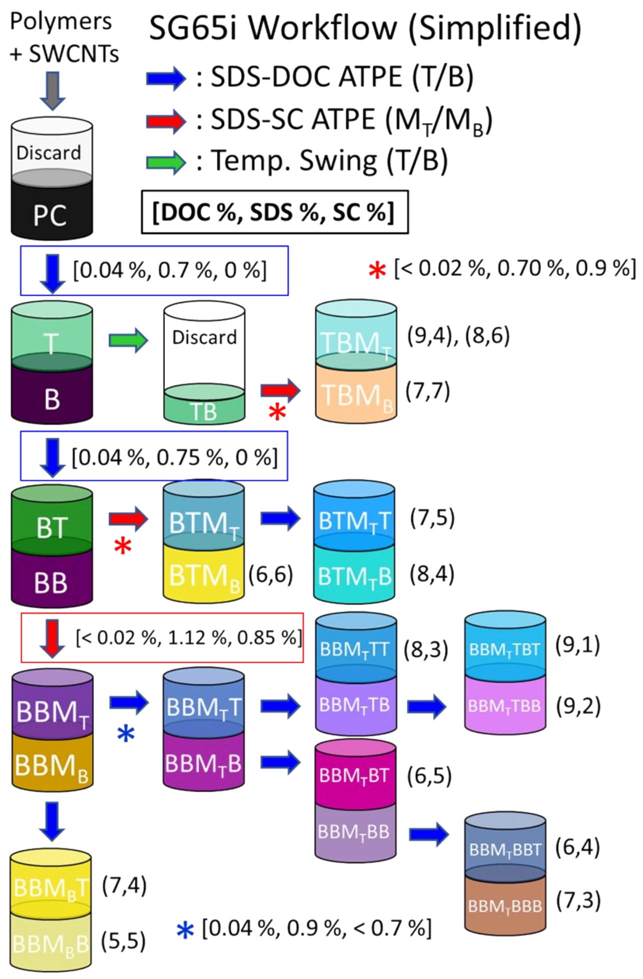

</td><td>
The diameter distribution of the SG65i includes a number of SWCNT (n,m) structures that are small enough such that there is significant “cross-talk” between S/M separation and diameter based partitioning if S/M is attempted at a single surfactant concentration condition.

The workflow diagram thus first separates the larger diameter (n,m) tail of the distribution, and then those (n,m)s that require less SDS for partition into the upper phase before S/M separation.

Note that this workflow is simplified, and the presence of additional (n,m) structures and enantiomers of various species will add complexity to reaching full isolation of some (n,m)s.

A typical oxidant level added during S/M ATPE for this material is 8 μL/mL to 10 μL/mL of the 1/100th concentration NaClO.
</td></tr>
</table>

Simplified Semiconducting (n,m) Separation	
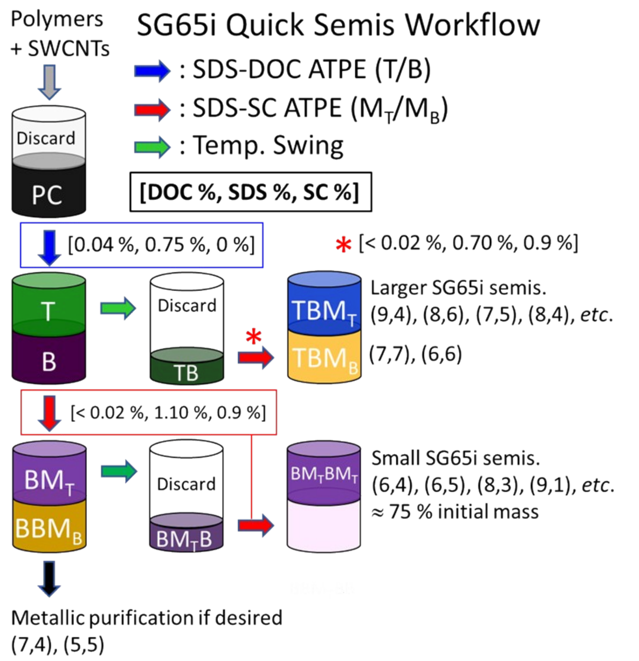	This workflow is partially optimized for the isolation of a high purity semiconducting population using a reduced number of ATPE steps and less polymer and surfactant consumption.<br>This workflow can be started from lower purity, dispersed/centrifuged or dispersed only SWCNTs (accepting contamination of metallic fractions with impurities). It is unclear what the optimal DOC concentration to both disperse and start the ATPE is, but it is likely in the 1.0 % - 1.5 % range.

B. High Pressure CO Disproportionation (HiPco) type

HiPco-type SWCNT populations are significantly more complex than CoMoCat materials to sort via ATPE as the diameter range, and consequently the number of (n,m)s is substantially larger. Additionally, the HiPco diameter range includes both outlier partitioning behavior SWCNT (n,m)s such as the (10,5), and (8,8), as well as small quantities of the (6,5), (6,4) and similar SWCNT species that added difficulty to the SG65i workflow. Significant spectral data for processing of HiPco SWCNTs *via* ATPE are presented in the SOI of Fagan *et al.*[4]

Normal Processing (Gradient Strategy)

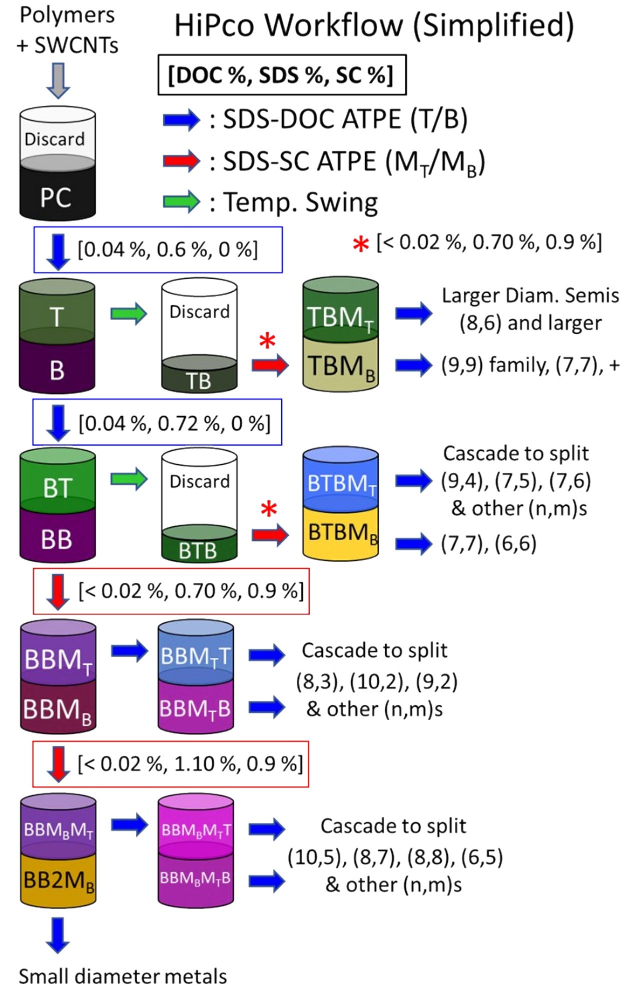

To attempt to simplify the separation of HiPco-type material it is advantageous to start with one to two diameter separation ATPE steps as shown in the workflow. This enables more applicable SDS and oxidant concentrations to be used for the different diameter subpopulations to reduce “cross-talk” that would otherwise result in co-mingled semiconducting and metallic SWCNTs in many populations.

The largest diameter population extracted can be treated similarly to the PT and LV workflow below. The intermediate diameters (shown here extracted as fraction BT) can be processed via a straight forward multistage cascade (although several of the species have strong differences in the separation conditions of their enantiomers).

The remaining populations (BB…) contain both the smaller diameter SWCNTs similar to the SG65i separation as well as the outlier partitioning (n,m)s.[4] Application of S/M separation at two different SDS concentrations and multistage diameter-structure ATPE can however resolve the individual species.

A typical oxidant level added during S/M ATPE for this material is 6 μL/mL to 8 μL/mL of the 1/100th concentration NaClO.

C. Plasma Torch (PT) or Laser Vaporization (LV)

These SWCNT populations tend to have a larger average diameter and narrower distribution than most HiPco populations that we have worked with, but a smaller average diameter than most electric arc (EA) synthesis populations. Substantial spectroscopy and other results were demonstrated in Fagan *et al.*[4]

<table>
<tr><th>Normal Processing (S/M then Gradient Strategy)</th><th></th></tr>
<tr><td>
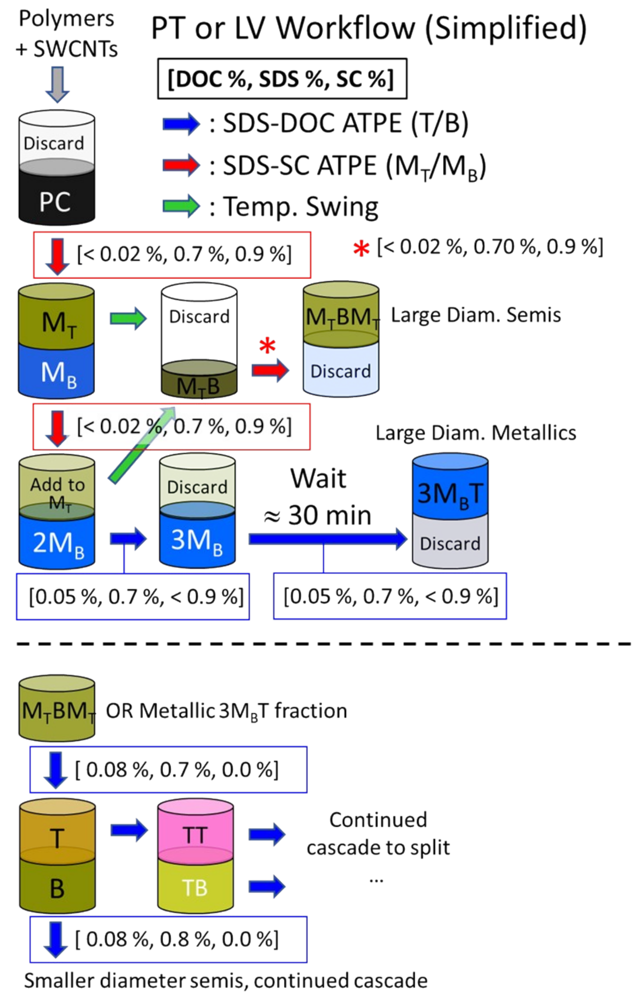

</td><td>
These SWCNT populations respond with high uniformity during S/M separation in our experience. Thus it is highly advantageous to first separate the semiconducting (n,m)s from the metallic species before (n,m) separation.

To improve diameter resolution the use of a somewhat increased DOC concentration during structure-diameter separation, as identified in Fagan et al.,[4] is recommended. Ease of isolation of particular (n,m) structures varies with these SWCNTs, particularly due to abundance differences, but separation of single (n,m) structures by hand is feasible.[4] One challenge, however, is that E22 optical transitions are near the edge of human eye sensitivity for some nanotubes in the population, making discrimination of a change more difficult or varying depending upon a person's individual color sensitivity.

A typical oxidant level added during S/M ATPE for this material is 5 μL/mL to 7 μL/mL of the 1/100th concentration NaClO.
</td></tr>
</table>

D. Electric Arc (EA) Synthesis

The average diameter of an EA population is in our experience around 1.5 nm, although it can be somewhat smaller or larger depending on the synthesis conditions. This larger average diameter likely includes an increased number of (n,m) structures than smaller average diameter populations simply due to the increased number of combinatorial (n,m) possibilities. This factor, and the spectral congestion of the E33 optical transitions (E22 transitions are outside the range of human eye sensitivity) increase the difficulty in by hand multistage structure-diameter ATPE. The population, however, responds well to S/M separation and is readily separated by that vector. Substantial spectroscopy and other results were demonstrated in Fagan *et al.*[4]

Normal Processing (S/M then Gradient Strategy)	
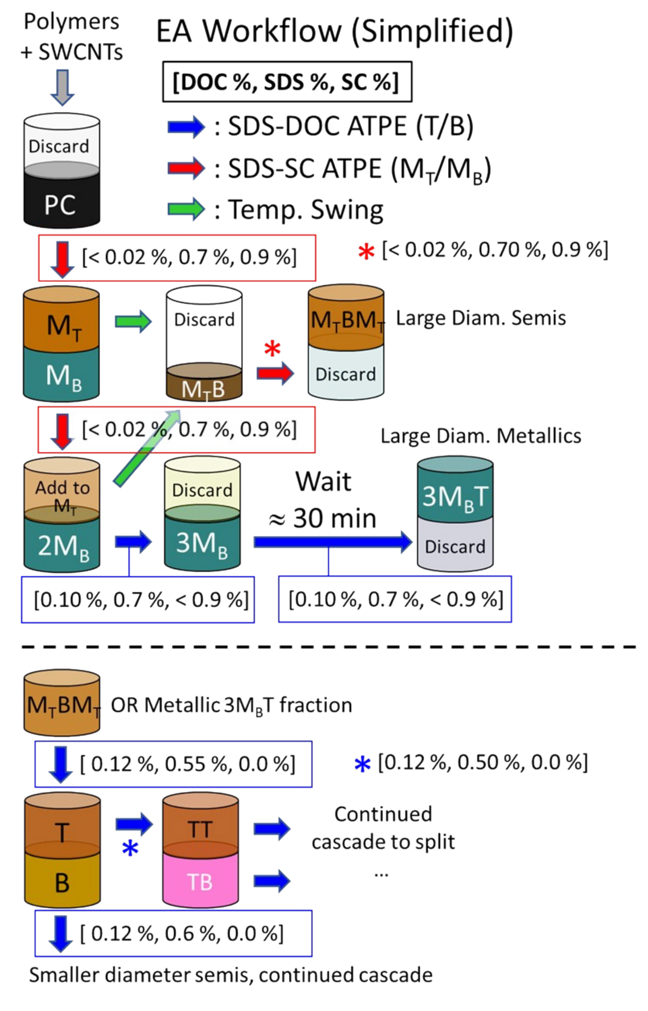 	These SWCNT populations respond with high uniformity during S/M separation in our experience. Thus it is highly advantageous to first separate the semiconducting (n,m)s from the metallic species before (n,m) separation.<br>After S/M separation, there are still many (n,m) structures in each population, with the most populous species estimated as comprising ≈ (1 to 4) % (number basis) in abundance. Thus, significant quantities of SWCNTs must be sorted to isolate many (n,m)s at an appreciable concentration. Additionally, while isolation of individual (n,m)s appears feasible by hand separation, identification of separation conditions that split populations along a different manner than DOS-SDS competition would dramatically simplify separation. One such strategy is the utilization of different oxidant concentrations to modulate ATPE partitioning on the basis of bandgap as demonstrated by Streit *et al.*,[6] additional strategies will be forthcoming as well.<br>A typical oxidant level added during S/M ATPE for this material is 5 μL/mL to 7 μL/mL of the 1/100th

<table>
<tr><td></td><td>concentration NaClO.</td></tr>
</table>

E. Floating Catalytic Decomposition (FCCD)

SWCNTs produced by this method have a broad diameter distribution centered at greater than 1.7 nm. The diameter distribution is so broad that the transitions add together to generate an absorbance spectrum that can appear to be only a decaying baseline, but it is really comprised of the sum of many transitions occurring across a broad range of wavelengths.

<table>
<tr><th>Normal Processing (S/M then Gradient Strategy)</th><th></th></tr>
<tr><td>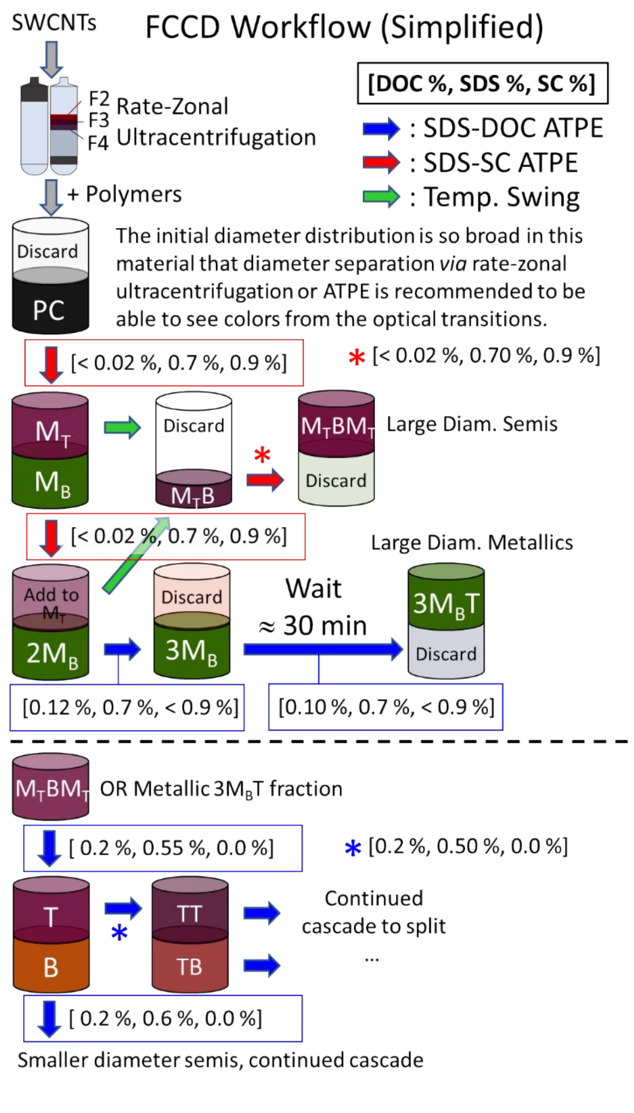
</td>
<td>Although it is desirable to first semiconducting-metallic sort these SWCNTs, similar to LV, PT, or EA SWCNTs, the broad diameter distribution means colors will be muddied, and a diameter distribution by either rate-zonal ultracentrifugation or an ATPE step is advised to enable visualization of the colors and reduced band gap variation during S/M separation.

The schematic on the left is for the middle (F3) portion of the diameter distribution. Note the inversion of the colors of the semiconducting and metallic SWCNTs from the smaller diameter populations of HiPco SWCNTs.

Due to the large average diameter, and thus many (n,m) structures, in the population distribution, multistep hand separation is challenging for these SWCNTs. Automation or tailored surfactant mixtures may be necessary for non-heroic isolation of specific (n,m) structures.

A typical oxidant level added during S/M ATPE for this material is 4 μL/mL to 6 μL/mL of the 1/100th concentration NaClO.</td></tr>
</table>

Date / /

Pg #

Cool

T

Name

Cool

T

Color:

Color:

To diam
To M/S
Collapse

To diam
To M/S
Collapse

#

Name

Cool

T

Color:

Color:

To diam
To M/S
Collapse

To diam
To M/S
Collapse

#

Name

Cool

T

Color:

Color:

To diam
To M/S
Collapse

To diam
To M/S
Collapse

#

Name

Cool

T

Color:

Color:

To diam
To M/S
Collapse

To diam
To M/S
Collapse

#

Name

Cool

T

Color:

Color:

To diam
To M/S
Collapse

To diam
To M/S
Collapse

ferences

[1] C. Y. Khripin, J. A. Fagan, M. Zheng Spontaneous Partition of Carbon Nanotubes in Polymer-Modified Aqueous Phases. *J. Am. Chem. Soc.* 2013, **135**, 6822–6825.
[2] J. Campo, Y. Piao, S. Lam, C. M. Stafford, J. K. Streit, J. R. Simpson, A. R. Hight Walker, J. A. Fagan Enhancing Single-Wall Carbon Nanotube Properties through Controlled Endohedral Filling. *Nanoscale Horizons*, 2016, **1**, 317–324.
[3] H. Gui, J. K. Streit, J. A. Fagan, A. R. H. Walker, C. Zhou, M. Zheng Redox Sorting of Carbon Nanotubes. *Nano Lett.* 2015, **15**, 1642–1646.
[4] J. A. Fagan, C. Y. Khripin, C. Silvera Batista, J. R. Simpson, E. H. Hároz, A. R. Hight Walker, M. Zheng Isolation of Specific Small-Diameter Single-Wall Carbon Nanotube Species via Aqueous Two-Phase Extraction. *Adv. Mater.* 2014, **26**, 2800–2804.
[5] J. A. Fagan, E. H. Hároz, R. Ihly, H. Gui, J. L. Blackburn, J. R. Simpson, S. Lam, A. R. Hight Walker, S. K. Doorn, M. Zheng Isolation of >1 nm Diameter Single-Wall Carbon Nanotube Species Using Aqueous Two-Phase Extraction. *ACS Nano* 2015, **9**, 5377–5390.
[6] J. K. Streit, S. Lam, Y. Piao, A. R. Hight Walker, J. A. Fagan, M. Zheng Separation of Double-Wall Carbon Nanotubes by Electronic Type and Diameter. *Nanoscale* 2017, **9**, 2531–2540.
